# Anodic oxidation of bisamides from diaminoalkanes by constant current electrolysis

**DOI:** 10.3762/bjoc.14.72

**Published:** 2018-04-16

**Authors:** Tatiana Golub, James Y Becker

**Affiliations:** 1 Department of Chemistry, Ben-Gurion University of the Negev, Beer Sheva 84105, Israel

**Keywords:** anodic oxidation, bisamides, constant current electrolysis, methoxylation

## Abstract

In general, bisamides derived from diamines and involving 3 and 4 methylene groups as spacers between the two amide functionalities behave similar to monoamides upon anodic oxidation in methanol/LiClO_4_ because both types undergo majorly mono- and dimethoxylations at the α-position to the N atom. However, in cases where the spacer contains two methylene groups only the anodic process leads mostly to CH_2_–CH_2_ bond cleavage to afford products of type RCONHCH_2_OCH_3_. Moreover, upon replacing LiClO_4_ with Et_4_NBF_4_ an additional fragmentation type of product was generated from the latter amides, namely RCONHCHO. Also, the anodic process was found to be more efficient with C felt as the anode, and in a mixture of 1:1 methanol/acetonitrile co-solvents.

## Introduction

**Scheme 1:**
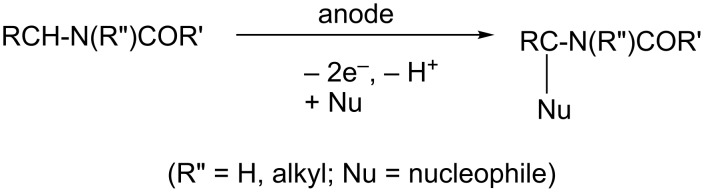
Anodic oxidation of amides.

**Scheme 2:**
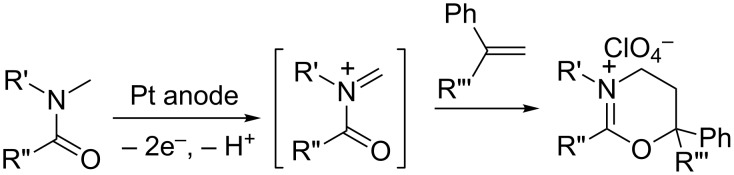
Anodic oxidation of an amide in the presence of alkene.

**Scheme 3:**
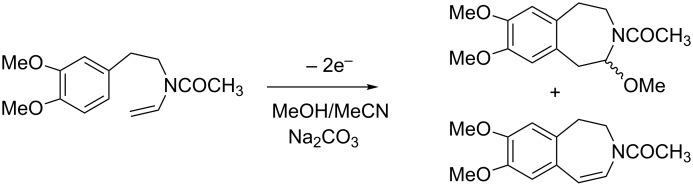
Intramolecular cyclization via anodic oxidation of an eneamide.

It is well known that the anodic oxidation of amides involving a hydrogen atom at the α-position to the N atom could undergo alkoxylation, carboxylation and hydroxylation at this position [1–5] (Scheme 1). It has been found that anodic methoxylation of amides (and carbamates) can be utilized to form new carbon–carbon bonds [6–7] (Scheme 2). Furthermore, this anodic route could also be important from a synthetic point of view, affording ring-expansion [8–10] and annulation of rings [1,11–12] (Scheme 3).

**Scheme 4:**
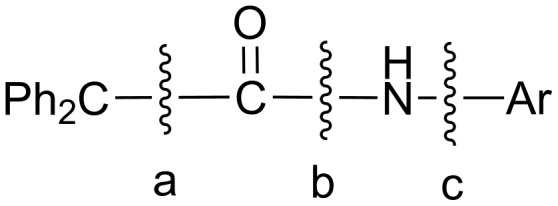
Anodic bond cleavages in amides of type Ph_2_CHCONHAr.

Interestingly, in the case of anodic oxidation of aromatic amides of type Ph_2_CHCONHAr, where no hydrogen atom is present at the α-position to the N atom, they undergo three types of bond cleavages (instead of the common substitution) [13] (Scheme 4).

**Scheme 5:**
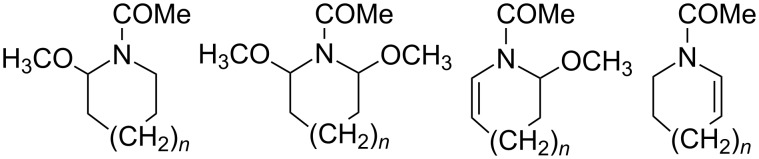
Type of products obtained (*n* = 0, 1, 2).

Previously we investigated [14] the effect of the ring size (5-, 6-, and 7-membered) and the nature of the supporting electrolyte on the anodic methoxylation of *N*-acylazacycloalkanes at the α-position to the nitrogen. The outcome revealed the formation of four types of products of which two involve saturated and two unsaturated cyclic amides (Scheme 5).

The selectivity of the anodic process was found to be highly dependent on the electrolyte used, and to a lesser extent, on the substrate concentration. Notably the importance of the former parameter in electrolysis has been well documented [15–16].

**Scheme 6:**
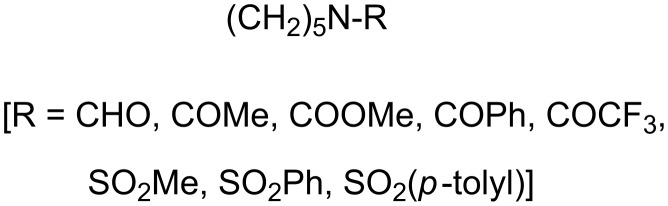
Synthesized cyclic *N*-acyl and *N*-sulfonyl piperidines for electrolysis.

More recently the effect of the functional group attached to the N atom in various piperidine derivatives (Scheme 6) on the anodic oxidation of 'cyclic amides' was explored in methanol under different experimental conditions [17]. The results indicate that this type of amides mostly undergo mono- and dimethoxylation at the α and α'-positions to the N atom. It was also found that the relative ratio among products was strongly dependent on the nature of the supporting electrolyte, the anode material and the substituent group attached to the N atom.

Amides and polyamides have been found as key units in many biologically active and pharmaceutical compounds. For instance, symmetrical and unsymmetrical bisamides derived from diamines are significant components as structural subunits for the construction of peptidomimetric frameworks [18] and as lubricants [19]. To the best of our knowledge nothing has been known so far about electrochemical properties of α,ω-bisamides derived from α,ω-diaminoalkanes. However, notably that the bisamide 3,5-diaza-2,6-heptanedione was obtained from *N*-methylacetamide by electrolysis on a Pt anode in water [20].

**Scheme 7:**
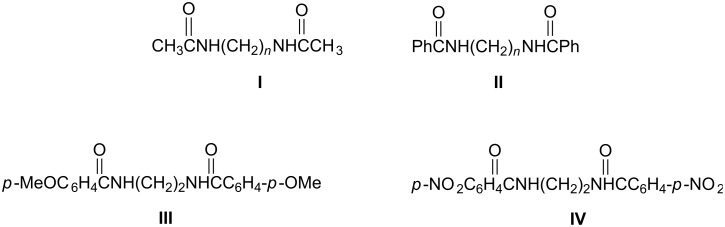
Type of bisamides (derived from diamines) studied (*n* = 2, 3, 4).

The present work describes the electrochemical behavior of eight synthesized bisamides (from diamines, Scheme 7) and the outcome from their preparative electrolysis (at constant current) under different experimental conditions, as a function of the length of spacer between the two amide functionalities.

## Results and Discussion

The electrochemical properties of the bisamides described in Scheme 6 were studied by cyclic voltammetry and all of their redox potentials were found to be irreversible in acetonitrile. Their first oxidation potentials (in the range of 2.1–2.35 V vs Ag/AgCl) are summarized in Table 1. The first and second columns indicate that the longer the spacer the higher the oxidation potential. Also it is not surprising that the derivative with EWG (**IV**) is more difficult to oxidize than that with EDG (**III**). All bisamides derivatives exhibit one irreversible cathodic wave (not shown, at −2.2 to −2.4 V).

**Table 1:** Oxidation potentials of α,ω-bisamides (Scheme 7) measured by cyclic voltammetry^a^.

entry	Ep_(ox)_ (V)			

	**I**	**II**	**III**	**IV**
	
*n* = 2	2.10	2.13	2.17	2.33
*n* = 3	2.23	2.28		
*n* = 4	2.27	2.35		

^a^In CH_3_CN/0.1 M LiClO_4_; potentials are quoted versus Ag/AgCl reference electrode. Working electrode: glassy carbon disk (1.5 mm in diameter). Auxiliary electrode: a Pt wire.

**Scheme 8:**
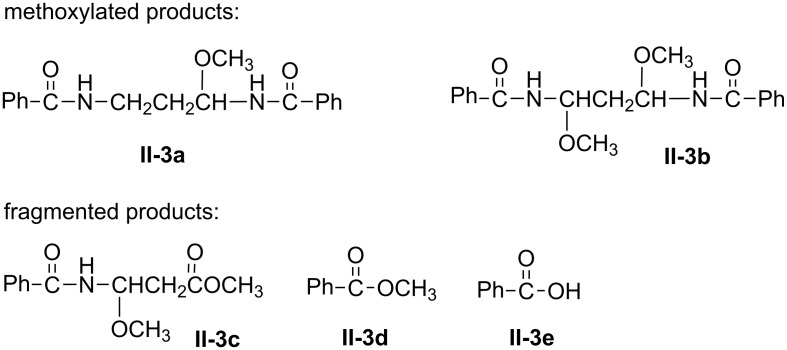
Type of products obtained from anodic oxidation of **II**-**3**.

Constant current electrolysis (CCE) at a current density of 20 mA/cm^2^ was carried out for all of the above synthesized bisamides under various experimental conditions, using different supporting electrolytes, anodes, and electricity consumption. Bisamide **II** (*n* = 3, will be designated as **II-3** hereafter) was arbitrarily chosen as a model compound for initial electrochemical studies. The spectrum of products obtained is described in Scheme 8. Except for the expected monomethoxylated **II-3a** and dimethoxylated **II**-**3b** products, fragmentation products (**II-3c**, **3d**, **3e**) were observed too.

Table 2 below summarizes the type of products and their relative ratios obtained from initial electrochemical oxidation of **II-3** under various experimental conditions. It appears that the selectivity and efficiency of the anodic process depends on both the anode material and electricity consumption (F/mol). Thus the oxidation of **II-3** on a C anode (Table 2, entries 1 and 2) is quite selective providing mostly mono-**II-3a** and dimethoxy- **II-3b** products in addition to ≈10% of methyl benzoate (**II-3d**) as a fragment. Notably that 20–30% of unreacted substrate remains. An increase in electricity consumption (entries 2 vs 1) promotes the formation of the dimethoxy product **II-3b** over the monomethoxy one **II-3a**.

**Table 2:** The effects of anode material and electricity consumption on the results of anodic oxidation of substrate **II-3** in MeOH/LiClO_4_^a^.

entry	F/mol	anode material	products					unreacted substrate

			**II-3a**	**II-3b**	**II-3c**	methyl benzoate (**II-3d**)	benzoic acid (**II-3e**)	
				
1	5	C	40	18	–	12	–	30
2	10	C	27	40	5	9	–	19
3	5	Pt	26	8	–	21	10	35
4	10	Pt	20	40	5	5	20	10
5^b^	10	GC	43	12	–	5	3	36
6^c^	10	PbO_2_	–	–	–	–	–	90
7^d^	10	DSA	–	–	–	–	–	≈100

^a^Yield of products was determined by ^1^H NMR relative integration. ^b^GC = glassy carbon. ^c^Unidentified products (≈10%). ^d^DSA = dimensionally stable anode, coated with oxides of Ru and Ir.

In comparison to the above results, oxidation of **II-3** on a Pt anode (Table 2, entries 3 and 4) affords similar products but with less selectivity because of the formation of an additional fragmentation product, benzoic acid (**II**-**3e**) in 10–20% yield. Other anodes were tested as well (Table 2, entries 5–7) at an electricity consumption of 10 F/mol. It appears that a GC anode favors the formation of the monomethoxy product **II-3a** whereas the anodes of PbO_2_ and DSA are the worst because most of the substrate remained unreacted. Apparently at these two anodes the oxidation of the solvent methanol prevails.

**Scheme 9:**
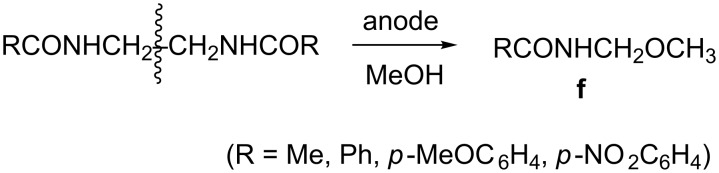
Anodic splitting of C–C bond in bisamides in the presence of LiClO_4_ electrolyte.

Based on the results in Table 2 in which a C anode afforded better selectivity and efficiency compared to the other anodes studied, all other substrates outlined in Scheme 7 were oxidized at this anode and under the same conditions (namely, in methanol/LiClO_4_, at 20 mA/cm^2^, and with electricity consumption of 10 F/mol). The results are shown in Table 3. It appears that except for entries 5 and 6, the amounts of unreacted starting materials are considerably high (60–70%), indicating that the reaction is far from being efficient under these conditions, presumably because of favorable oxidation of the solvent methanol. Another reason for this observation could stem partially from the limited solubility of some of the substrates in this solvent (e.g., **II-2**, **III-2** and **IV-2**). The yield of total products is in the range of 30–80%, depending on the nature of the substituent attached to the carbonyl moiety, roughly in the order of: Ph > CH_3_ > *p*-MeOC_6_H_4_, *p*-NO_2_C_6_H_4_. In terms of type of products, they are mostly analogous to those described in Scheme 8 for **II-3**. However, it is obvious that in the case of anodic oxidation of substrates with *n* = 2, an additional new fragmented product (type **f**) was formed due to CH_2_–CH_2_ bond cleavage, which is exclusive for this type of bisamides (Scheme 9).

**Table 3:** Results of anodic oxidation of all substrates on a C rod anode in MeOH/LiClO_4_. (10 F/mol; 20 mA/cm^2^)^a^.

entry	substrate	monomethoxy type '**a'**	dimethoxy type '**b**'	methoxylated amide type '**c**'	methyl benzoate '**d**'	RCONHCH_2_OCH_3_ '**f**'	unreacted substrate

1	**I-2** (*n* = 2)	10	–	–	–	25	65
2	**I-3** (*n* = 3)	30	10	–	–	–	60
3	**I-4** (*n* = 4)	5	30	–	–	–	65
4	**II-2**	9	–	–	5	23	63
5	**II-3**	27	40	5	9	–	19
6	**II-4**	26	27	–	17	–	30
7	**III-2**	15	–	–	–	15	70
8^b^	**IV-2**	–	–	–	–	10	65

^a^Relative yields of all products were determined by ^1^H NMR integration. Analogous type of products (**a**–**d**) are described in Scheme 7. ^b^PhCONH_2_ was obtained in 10% yield along with 25% of unidentified products.

**Scheme 10:**
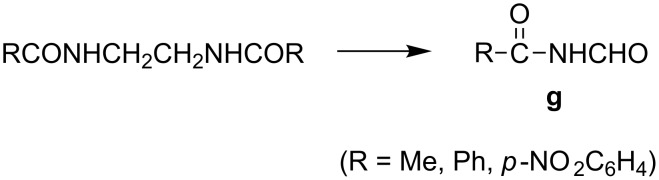
Anodic splitting of C–C bond in bisamides in the presence of Et_4_NBF_4_ electrolyte.

Obviously the fact that considerable amounts of starting materials were left unreacted (in most cases, except for Table 3, entries 5 and 6) has been dissatisfying and therefore, prompted us to change some parameters. At first, the LiClO_4_ electrolyte was replaced by Et_4_NBF_4_ and the results are shown in Table 4. Clearly the bisamides with *n* = 2 afforded now, in addition to **f**, new fragmented products, aldehydes of type **g** (Scheme 10).

In addition, the relative yield of the fragmented products (**f** and **g,** both derived exclusively from bisamides with *n* = 2) increased considerably in the presence of this electrolyte. However, again, the amounts of unreacted starting materials were still significant in some cases.

**Table 4:** Results of preparative electrolysis of selected bisamides in MeOH/Et_4_NBF_4_ (at C rod anode, 10F, 20 mA/cm^2^)^a^.

entry	substrate	monomethoxy type '**a**'	dimethoxy type '**b**'	RCONHCH_2_OCH_3_ '**f**'	RCONHC(O)H '**g**'	unreacted substrate

1	**I-2**	10	–	51	26	13
2	**I-3**	10	10	–	–	80
3	**I-4**	13	–	–	–	87
4	**II-2**	–	–	69	17	14
5	**III-2** (*p*-OMe)	–	–	23	–	77
6	**IV-2** (*p*-NO_2_)	–	–	51	31	18

^a^Yields were determined by ^1^H NMR relative integration. Analogous type of products **a**,**b** are described in Scheme 7.

The pronounced difference between the results obtained by the two electrolytes, namely affording '**f**' with LiClO_4_, and '**g**' in addition to '**f**' with Et_4_NBF_4_, could stem from the different composition of the solution at the electrode surface (caused by different solvation of the electrolyte anion) than that of the bulk of the solution. Such a phenomenon was discussed in the literature previously [15–16,21]. For instance, Nyberg [16] already demonstrated the effect of ClO_4_^−^ vs BF_4_^−^ in the anodic oxidation of hexamethylbenzene in aqueous acetonitrile, and proposed that tetraflouroborate anion preferentially brought water into the anode surface giving high yield of ArCH_2_OH (compared to ArCH_2_NHCOMe with perchlorate). Therefore also in our case, the electro generated carbocation intermediate formed (RCONHCH_2_^+^) could meet with methanol (to form '**f**') or water (to form '**g**') preferentially at the electrode surface, dictating the ratio between products '**f**' and '**g**'.

In order to increase the solubility of the substrates with a limited one a mixture of MeOH/MeCN (1:1) was used, and in parallel, the C rod anode was replaced with a C felt (that has a considerable larger surface area) in attempts to improve both efficiency and selectivity. The results are described in Table 5 and they show a pronounced difference compared to the ones in Table 4 because product yields are higher now (Table 5, entries 1, 4 and 5 show almost completion) even after consuming only 5 F/mol. In addition, although the spectrum of major products is similar in both tables, the weight of monomethoxylated products (51–56%, entries 2 and 5) and fragmented ones (of type **f**, 85–95%, entries 1 and 4) increased at the expense of consumed starting material (in all entries except for entry 8).

**Table 5:** Results^a^ of anodic oxidation of bisamides on a C felt anode in MeOH/MeCN (1:1)/LiClO_4_; 20 mA/cm^2^; 5 F/mol).

entry	substrate	monomethoxy type '**a**'	dimethoxy type '**b**'	ester type '**d**'	benzoic acid '**e**'	RCONHCH_2_OCH_3_ '**f**'	unreacted substrate

1	**I-2**	5	–	–	–	95	–
2	**I-3**	56	7	–	–	–^b^	34
3	**I-4**	20	30	–	–	–	50
4	**II-2**	–	–	15	–	85	–
5	**II-3**	51	24	8	5	(**h**)^c^	5
6	**II-4**	19	22	16	–	–	43
7	**III-2**	10	–	5	–	25	60
8	**IV-2**	–	–	5	5	5	85

^a^Yield are determined by ^1^H NMR relative integration. Analogous type of products (type **a**, **b**, **d**, **e**) are described in Scheme 7. ^b^Aldehyde (3%): MeCONHCH_2_CH_2_CHO (from, **I-3**) [22]. ^c^Unsaturated bisamide (7%): PhCONHCH_2_CH=CHNHCOPh (**II-3h**).

Previously (Table 3 vs Table 4) we observed a marked difference in results upon replacing LiClO_4_ with Et_4_NBF_4_. Whereas in the former case mono- and dimethoxylated products were predominant, fragmentation products of type **f** and **g** became major in the latter case. Based on these observations a further attempt to improve the results outlined in Table 5 was conducted by employing similar conditions except for using Et_4_NBF_4_ (instead of LiClO_4_) this time. Some selected substrates from Table 5 that left a considerable amount of unreacted starting material, namely **I-4, II-4, III-2** and **IV-2**, were chosen to be reoxidized under these modified conditions. The outcome described in Table 6 indicates that this approach was useful for one substrate only, **II**-**4**.

**Table 6:** Results of anodic oxidation of selected substrates on C felt anode in MeOH/MeCN (1:1)/Et_4_NBF_4_; 20 mA/cm^2^; 5 F/mol.

entry	substrate	monomethoxy type '**a**'	dimethoxy type '**b**'	ester type '**d**'	fragmented products	unreacted substrate

1	**I-4**	10	–	–	MeCONHCHO (**I-4g**, 5%)	85
2	**II-4**	12	74	8	–	6
3	**III-2** (*p*-OMe)	–	–	15	ArCONHCH_2_OCH_3_ (**III-2f,** 10%)	75
4	**IV-2** (*p*-NO_2_)	–	–	31	–	69

## Mechanism

**Scheme 11:**
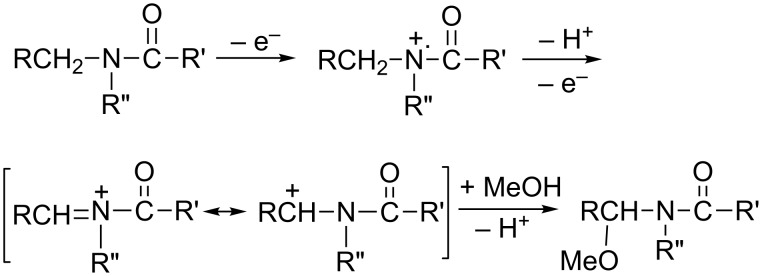
A suggested mechanism for anodic methoxylation of amides.

A mechanism of formation of mono- and dimethoxylated amides is well-documented in the published literature [1–6,14,17,23]. It is generally accepted that the initial electron transfer forms an iminium cation radical followed by deprotonation and further oxidation to generate an iminium ion/carbocation that undergoes methoxylation in methanol, as described in Scheme 11.

**Scheme 12:**
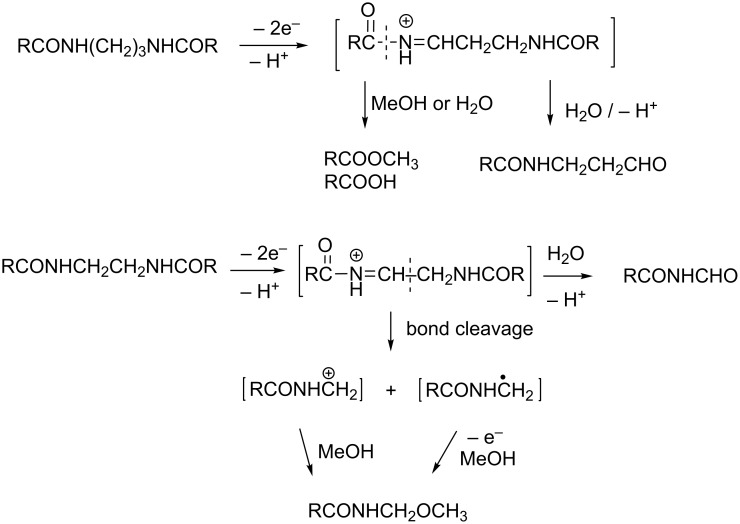
Mechanisms of formation of fragmentation products.

Plausible mechanisms for the formation of various fragmentation products are described in Scheme 12.

Actually whenever benzoic acid or methyl benzoate were formed (top of Scheme 12), the corresponding aldehyde from the other part of the molecule was detected too and fully characterized.

## Conclusion

In general, bisamides derived from diamines and involving 3 and 4 methylene groups as spacers between the two amide functionalities behave similary to monoamides upon anodic oxidation in methanol/LiCiO_4_ because both types undergo majorly mono- and dimethoxylations at the α-position to the N atom. However, in cases where the spacer contains two methylene groups only the anodic process leads mostly to CH_2_–CH_2_ bond cleavage to afford products of type RCONHCH_2_OCH_3_. Moreover, upon replacing LiClO_4_ with Et_4_NBF_4_ an additional fragmentation type of product was generated from the latter amides, namely RCONHCHO. Also, the anodic process was found to be more efficient with C felt as the anode, and in a mixture of 1:1 methanol/acetonitrile co-solvents.

## Experimental

### Materials

Reagents, electrolytes and solvents (all analytical grade) were supplied by various vendors, as mentioned in [23].

### Preparation of bisamides **I**–**III**

All types of bisamides were prepared according to our own procedure by reacting the corresponding diamines (commercially available) with acetic anhydride, or benzoyl chloride or 4-methoxybenzoyl chloride. In a typical experiment, 30 mmol of a diamine (ethylenediamide, 1,3-diaminopropane or 1,4-diaminobutane) were introduced into a 500 mL Erlenmeyer flask with 50 mL of DCM and 20 mL of saturated aqueous bicarbonate solution. Then 70 mmol of acetic anhydride (or benzoyl chloride or 4-methoxybenzoyl chloride) were added dropwise by a separatory funnel to the diamine solution while stirring by a magnetic stirrer. Then the reaction mixture was filtered under vacuum and the solid residue was recrystallized from a mixture of ethyl acetate and water (9:1). The resulting white precipitate (except for the yellowish one derived from *p*-nitrobenzene derivative) was dried, weighed and verified by NMR spectra. The isolated yields of the bisamides were around 72–86%.

### General methods

Instruments used in this study for ^1^H NMR and ^13^C NMR measurements, mass and IR spectra, high-resolution mass analyses, and cyclic voltammetry were described in [23].

Thin-layer chromatography (TLC) was carried out on aluminum sheets coated with aluminum oxide 60 F_254_ and silica gel 60 F_254_. Retention time was evaluated by UV (for amides with benzene ring) or by using a general purpose stain of cerium molybdate [containing a mixture of Ce(NH_4_)_2_(NO_3_)_6_ - (NH_4_)_6_Mo_7_·4H_2_O in H_2_SO_4_] for amides of type **I**. Preparative TLC was carried out by using 20 × 20 cm of glass plates coated with silica gel 60 F_254_. Evaporation of solvents was performed at reduced pressure using a rotary evaporator.

### Constant current electrolysis

Constant current electrolysis at preparative scale was performed at constant currents using a PAR Potentiostat/Galvanostat Model 273A, and a beaker-type undivided cell equipped with a C rod, C felt, PbO_2_, GC, DSA or a Pt foil (immersed area of ≈5 cm^2^) as the anode, and a Pt foil as the cathode. In a typical electrolysis α,ω-bisamides (1 mmol) were dissolved in methanol (or 1:1 methanol/acetonitrile, 25 mL) containing 0.1 M supporting electrolytes. Electrolysis took place at room temperature with a current density of 20 mA_/_cm^2^ and was terminated after the desired consumption of electricity was passed (Tables 2–5). Then the reaction mixture was concentrated by rotary evaporator till all the solvents evaporated. The relative yield of products was determined by ^1^H NMR integration. Notably this procedure of analyzing a mixture of products simultaneously and successfully is based on prior separation and characterization of the individual products in the mixture. Their previous separation was carried out either by silica gel column chromatography or preparative coated glass plates, using different mixtures of ethyl acetate (20–50%)/hexane or acetone/ethyl acetate, as eluent. Also since some of the products undergo facile hydrolysis/decomposition it is suggested that the analysis will be done immediately after terminating the electrolysis.

### Characterization of products

See Supporting Information File 1 for spectral data and copies of ^1^H and ^13^C NMR spectra.

## Supporting Information

File 1 Spectral data and copies of ^1^H and ^13^C NMR spectra.
