# Supplementary material for: Anodic oxidation of bisamides from diaminoalkanes by constant current electrolysis
Source: Beilstein J Org Chem. 2018 Apr 16;14:861–8. doi: 10.3762/bjoc.14.72 (PMC5942381; doi:10.3762/bjoc.14.72)
Supplement: File 1 — Spectral data and copies of 1H and 13C NMR spectra. [file Beilstein_J_Org_Chem-14-861-s001.pdf]

Supporting Information

for

Anodic oxidation of bisamides from diaminoalkanes by constant current electrolysis

Tatiana Golub and James Y. Becker*

Address: Department of Chemistry, Ben-Gurion University of the Negev, Beer Sheva 84105, Israel

Email: James Y. Becker - becker@bgu.ac.il

*Corresponding author

Spectral data and copies of ^{1}H and ^{13}C NMR spectra.

Characterization of products

Products with marked references are known. The rest are new ones.

***N,N'*-(1-Methoxyethane-1,2-diyl)diacetamide (I-2a)**

IR (liquid): 1100, 1250, 1645, 2850, 2920, 2960, 3410; ^{1}H NMR ($CDCl_3$, 400 MHz) δ: 1.99 (s, 3H, $-CH_3$), 3.33 and 3.52 (2m, 2H, $-CH_2-$), 3.37 (s, 3H, $-OCH_3$), 5.87 and 6.45 (2m, 2H, NH), ^{13}C NMR ($CDCl_3$, 100 MHz) δ: 23.37 ($-CH_3$), 40.03 ($-CH_2-$), 53.91 and 56.19 ($-OCH_3$), 79.87 (-CH-), 171.62 (-CO). HRMS (ESI): calculated for $C_7H_{14}N_2O_3$+Na: 197.08966; found: 197.08961.

***N*-(Methoxymethyl)acetamide (I-2f)** [1]

IR (liquid): 1250, 1500, 1655, 2850, 2920, 2960, 3425; ^{1}H NMR ($(CD_3)_2CO$, 400MHz) δ: 1.95 (s, 3H, $-CH_3$), 3.22 (s, 3H, $-OCH_3$), 4.55 (d, 2H, -CH2-), 7.98 (brs, 1H, NH); ^{13}C NMR ($CDCl_3$, 100 MHz) δ: 23.53 (CH_3), 56.15 ($-OCH_3$), 71.55 (-CH-), 170.96 (CO). MS: m/z (%): 88 (M^+-15), 73.0, 58.9, 44.0. (100). HRMS (ESI): calculated for $C_4H_9NO_2$+H: 104.07061; found: 104.07045.

***N*-Formylacetamide (I-2g)** [2]

^{1}H NMR ($CDCl_3$, 400MHz) δ: 2.21 (s, 3H, -CH_3), 8.89 (brs, 1H, NH) 9.08 (d, 1H, CHO); ^{13}C NMR ($CDCl_3$, 100 MHz) δ: 23.85 (CH_3), 163.48 (CHO), 170.48 (CH_3CO).

***N,N'*-(1-Methoxypropane-1,3-diyl)diacetamide (I-3a)**

^{1}H NMR ($CDCl_3$, 400 MHz) δ: 1.81 (m, 2H, -CH_2-), 1.97, 2.01 (2s, 6H, -CH_3), 3.11 and 3.56 (2m, 2H, -CH_2), 3.38 (1s, 3H, -OCH_3), 5.19 (m, 1H, -CH-), 5.99 and 6.07 (2 br, 2H, NH), ^{13}C NMR ($CDCl_3$, 100 MHz) δ: 23.36 and 23.53 (-CH_3), 31.06 (-CH_2-), 56.49 (-OCH_3), 85.12 (-CH-), 171.70 (-CO). HRMS (ESI): calculated for $C_8H_{16}N_2O_3$+Na: 211.10531; found: 211.10516.

***N,N'*-(1,3-Dimethoxypropane-1,3-diyl)diacetamide (I-3b)**

^{1}H NMR ($CDCl_3$, 400 MHz) δ: 1.99 (m, 2H, -CH_2-), 1.94 (s, 6H, -CH_3), 3.40 (1s, 6H, -OCH_3), 5.29 (m, 2H, -CH-), 6.41 (m, 1H, NH), ^{13}C NMR ($CDCl_3$, 100 MHz) δ: 23.44 (-CH_3), 36.14 (-CH_2-), 55.94 (-OCH_3), 80.23 (-CH-), 170.66 (-CO). HRMS (ESI): calculated for $C_9H_{20}N_2O_4$+Na: 243.13153; found: 243.13148.

***N,N'*-(1-Methoxybutane-1,4-diyl)diacetamide (I-4a)**

^{1}H NMR ($(CD_3)_2CO$, 400 MHz) δ: 1.56-1.73 (2m, 4H, -CH_2-CH_2-), 1.95 and 1.97 (2s, 6H, -CH_3), 3.26 (m, 2H, -CH_2-), 3.31 (s, 3H, -OCH_3), 5.12 (m, 1H, -CH-), 7.67 and 8.10 (brd, 2H, NH), ^{13}C NMR (CD_3CN, 100 MHz) δ: 22.59 (-CH_2-), 23.26 (-CH_3), 31.76 (-CH_2-), 39.17 (-CH_2-) ,54.80 (-OCH_3), 83.56 (-CH-), 172.77 (-CO). HRMS (ESI): calculated for $C_9H_{18}N_2O_3$+Na: 225.12096; found: 225.12093.

***N,N'*-(1,4-Dimethoxybutane-1,4-diyl)diacetamide (I-4b)**

^{1}H NMR ($(CD_3)_2CO$, 400 MHz) δ: 1.49 (m, 4H, -CH_2-CH_2-), 1.99 (1s, 6H, CH_3), 3.27 (1s, 6H, -OCH_3), 5.25 (m, 2H, -CH-), 7.63 (brs, 2H, NH), ^{13}C NMR ($CDCl_3$, 100

MHz) δ: 23.41 (-CH_3), 26.91 (-CH_2-), 52.65 (-OCH_3), 83.43 (-CH-), 170.67 (-CO). HRMS (ESI): calculated for $C_{10}H_{20}N_2O_4$+Na: 255.13153; found: 255.13164.

***N,N'*-(1-Methoxyethane-1,2-diyl)dibenzamide (II-2a)**

^{1}H NMR ($CDCl_3$, 400 MHz) δ: 3.36 and 3.96 (2m, 2H, -CH_2-), 3.44 (s, 3H, -OCH_3), 5.52 (m, 1H, -CH-), 7.26-7.89 (10H, Ph), 6.80 and 6.98 (2m, 2H, NH), ^{13}C NMR ($(CD_3)_2CO$, 100 MHz) δ: 51.85 (-CH_2-), 55.98 (-OCH_3), 83.51 (-CH-), 128.18, 129.26, 132.43, 133.75 (Ph), 167.51 (-CO). HRMS (ESI): calculated for $C_{10}H_{20}N_2O_4$+Na: 321.12096; found: 321.11969.

***N*-(Methoxymethyl)benzamide (II-2f)** [3]

IR (liquid): 1180, 1300, 1540, 1658, 2825, 2915, 3000, 3335; ^{1}H NMR ($CDCl_3$, 400 MHz) δ: 3.40 (s, 3H, -OCH_3), 4.90 (d, 2H, -CH2-), 6.94 (brs, 1H, NH), 7.42-7.82 (5H, -Ph); ^{13}C NMR ($CDCl_3$, 100 MHz) δ: 56.31 (-OCH_3), 72.03 (-CH-), 127.22, 128.80, 132.10, 133.97 (-Ph), 168.15 (CO). MS: m/z (%): 150 (M^+-15), 134, 121, 105 (100), 91, 77, 51. HRMS (ESI): calculated for $C_9H_{11}NO_2$+H: 166.08626; found: 166.08614.

***N*-Formylbenzamide (II-2g)** [4]

^{1}H NMR ($CDCl_3$, 400 MHz) δ: 7.56-7.91 (5H, -Ph), 9.01 (brs, 1H, NH), 9.37 (d, 1H, CHO); ^{13}C NMR ($CDCl_3$, 100 MHz) δ: 127.91, 129.34, 134.12, 163.32 (CO). MS: m/z (%): 150 (M^+), 134, 121, 105 (100), 91, 77, 51.

***N,N'*-(1-Methoxypropane-1,3-diyl)dibenzamide (II-3a)**

^{1}H NMR ($(CD_3)_2CO$, 400 MHz) δ: 2.00-2.12 (2m, 2H, -CH_2-), 3.45 (1m, 2H, -CH_2-), 3.37 (1s, 3H, -OCH_3), 5.47 (m, 1H, -CH-), 8.10 (brd, 2H, NH), 7.47-7.91 (2m, 10H, Ph), ^{13}C NMR ($(CD_3)_2CO$, 100 MHz) δ: 35.70 (-CH_2-), 37.38 (-CH_2-), 55.84 (-OCH_3), 81.08 (-CH-), 127.94-135.23 (Ph), 167.67 (-CO). HRMS (ESI): calculated for $C_{18}H_{20}N_2O_3$+Na: 335.13661; found: 335.13605.

***N,N'*-(1,3-Dimethoxypropane-1,3-diyl)dibenzamide (II-3b)**

^{1}H NMR ($CDCl_3$, 400 MHz) δ: 2.10 - 2.26 (2m, 2H, -CH_2-), 3.34 and 3.53 (2s, 6H, -OCH_3), 5.69 (m, 2H, -CH-), 7.08 and 8.31 (2m, 2H, NH), 7.46-7.85 (10H, Ph), ^{13}C NMR ($CDCl_3$, 100 MHz) δ: 40.24 (-CH_2-), 55.26 (-OCH_3), 78.73 and 80.89 (-CH-), 127.1-133.99 (-Ph) 167.54 (-CO). HRMS (ESI): calculated for $C_{19}H_{22}N_2O_4$+Na: 365.14718; found: 365.14655.

Methyl 3-benzamido-3-methoxy)propanoate (II-3c)

^{1}H NMR ($CDCl_3$, 400 MHz) δ: 2.9 (qd, 2H, -CH_2-), 3.41 (1s, 3H, -OCH_3), 3.76 (1s, 3H, -OCH_3), 5.69 (m, 1H, -CH-), 7.75 (m, 1H, NH), 7.47-7.86 (10H, Ph). ^{13}C NMR (acetone-d_6, 100 MHz) δ: 40.68 (-CH_2-), 51.85 (-OCH_3), 55.98 (-OCH_3), 79.02 and 79.28 (-CH-), 128.18-133.75 (-Ph) 167.51 (CO), 170.92 (CO); MS: m/z (%): 222 (M^+-15), 205, 174, 146, 132, 105 (100), 91, 77, 51; HRMS (ESI): calculated for $C_{18}H_{20}N_2O_3$+Na: 335.13661; found: 335.13605.

***(E)-N,N'*-(Prop-1-ene-1,3-diyl)dibenzamide** (**II-3h**) (minor, 7%);

^{1}H NMR ($CDCl_3$, 400 MHz) δ: 4.28 (m, 2H, -CH_2-), 5.69 (q, 1H, -C*H*=CHNH), 6.78 (d, 1H, =C*H*NH), 8.44 (brs, 2H, NH), 7.53-7.88 (m, 10H, Ph); ^{13}C NMR ($CDCl_3$, 100 MHz) δ: 39.71, 100.54, 125.34, 125.34, 127.53, 128.78, 132.26, 134.25 (-C_6H_4), 163.67 (-CO), 167.61 (-CO).

***N,N'*-(1-Methoxybutane-1,4-diyl)dibenzamide (II-4a)**

^{1}H NMR ($(CD_3)_2CO$, 400 MHz) δ: 1.76-1.96 (2m, 4H, -CH-CH_2-CH_2-), 3.20 and 3,46 (2m, 2H, -CH_2-N), 3.28 (1s, 3H, -OCH_3), 5.24 (m, 1H, -CH-), 7.43-7.96 (2m, 10H, Ph), 7.87 and 8.15 (2m, 1H, NH), ^{13}C NMR ($(CD_3)_2CO$, 100 MHz) δ: 30.46 (CH_2), 31.84 (-CH_2-), 38.98 (-CH_2-), 53.79 (-OCH_3), 81.51 (-CH-), 127.27-134.23 (-Ph), 167.85 (-CO). HRMS (ESI): calculated for $C_{19}H_{22}N_2O_3$+Na: 349.15226; found: 349.15109.

N,N'-(1,4-Dimethoxybutane-1,4-diyl)dibenzamide (II-4b)

^{1}H NMR ($(CD_3)_2CO$, 400 MHz) δ: 1.74 - 1.94 (dt, 2H, $-CH_2-CH_2-$), 3.32 (1s, 6H, -OCH_3), 5.34 (m, 2H, -CH-), 7.85 (brd, 2H, NH), 7.44 - 7.976 (2m,10H, Ph), ^{13}C NMR ($(CD_3)_2CO$, 100 MHz) δ: 26.29 ($-CH_2-$), 55.62 ($-OCH_3$), 82.36 (-CH-), 128.28, 129.17, 131.78, 132.26 (-Ph), 167.38 (-CO). HRMS (ESI): calculated for $C_{20}H_{24}N_2O_4$+K: 395.13677; found: 395.13605.

N,N'-(1-Methoxyethane-1,2-diyl)bis(4-methoxybenzamide) (III-2a)

^{1}H NMR ($(CD_3)_2CO$, 400 MHz) δ: 3.33 (2m, 2H, $-CH_2-$), 3.46 (s, 3H, $-OCH_3$), 3.85 (s, 3H, $-OCH_3$), 4.74 (m, 1H, -CH-), 6.99 and 7.91 (dd, 8H, $-C_6H_4$), 8.31 (brs, 1H, NH); ^{13}C NMR ($(CD_3)_2CO$, 100 MHz) δ: 51.02 (CH_2), 55.66 ($CH-OCH_3$); 56.87 ($2OCH_3$), 87.29 (-CH-), 114.47, 127.95, 132.38, 164.01 ($-C_6H_4$), 168.39 (CO); HRMS (ESI): calculated for $C_{19}H_{22}N_2O_5$+Na: 381.14209; found: 381.14038.

4-Methoxy-*N*-(methoxymethyl)benzamide (III-2f)

IR (liquid): 1100, 1655, 2825, 2915, 3000, 3400; ^{1}H NMR ($CDCl_3$, 400 MHz) δ: 3.40 (s, 3H, $-OCH_3$), 3.86 (1s, 3H, OCH3), 4.90 (d, 2H, $-CH_2-$), 6.70 (brs, 1H, NH), 6.95 and 7.78 (dd, 4H, $-C_6H_4$); ^{13}C NMR ($CDCl_3$, 100 MHz) δ: 55.59 and 56.31 ($2OCH_3$), 72.02 (-CH-), 114.03, 129.10, 137.94, 161.41 (C_6H_4), 169.09 (-CO), MS: m/z (%): 195.0 (M^+), 181.0, 165.0 (100), 149.9, 122.0, 106.9, 79.1, 45. HRMS (ESI): calculated for $C_{10}H_{13}NO_3$+Na: 218.07876; found: 218.07858.

***N*-(Methoxymethyl)-4-nitrobenzamide (IV-2f)**

IR (liquid): 1250, 1655, 2850, 2930, 3360; ^{1}H NMR ($CDCl_3$, 400 MHz) δ: 3.43 (s, 3H, $-OCH_3$), 4.93 (d, 2H, $-CH_2-$), 6.82 (brs, 1H, NH), 7.98 and 8.32 (dd, 4H, C_6H_4); ^{13}C NMR ($CDCl_3$, 100 MHz) δ: 56.21, 73.59 (-CH-), 124.13, 130.30, 140.47, 150.79 (C_6H_4), 169.27 (-CO). HRMS (ESI): calculated for $C_9H_{10}N_2O_4$+H: 211.07188; found: 211.07127.

N-Formyl-4-nitrobenzamide (IV-2g)

^{1}H NMR ((CD_3)$_2$CO, 400 MHz) δ: 9.09 (d, 1H, -CHO), 8.33 (brs, 1H, NH), 8.20 and 8.33 (dd, 4H, -C_6H_4); ^{13}C NMR ((CD_3)$_2$CO, 100 MHz) δ: 124.03, 129.62, 139.22, 152.55 (-C_6H_4), 161.42 (-CO), 168.31 (-CO). HRMS (ESI): calculated for $C_8H_6N_2O_4$+Na: 217.02253; found: 217.02204.

^{1}H NMR and ^{13}C NMR spectra

***N,N'*-(1-Methoxyethane-1,2-diyl)diacetamide (I-2a)**

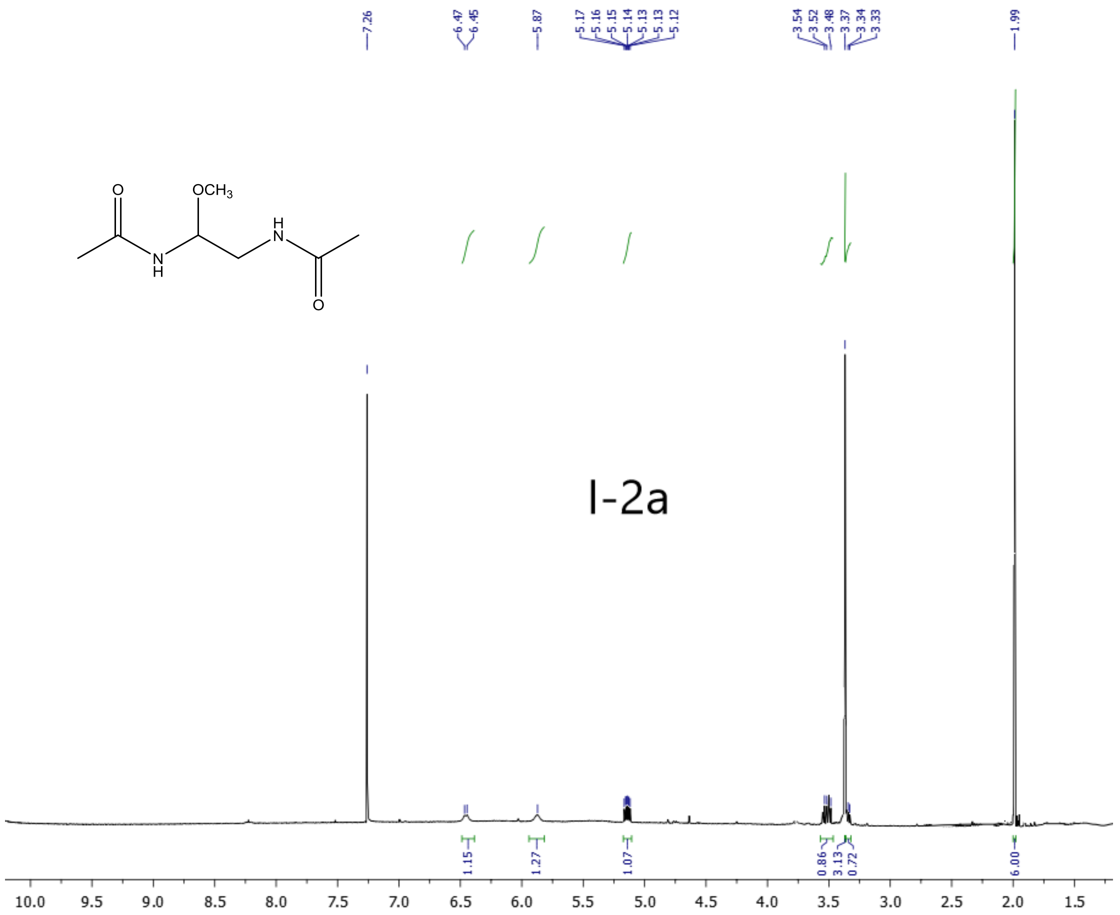

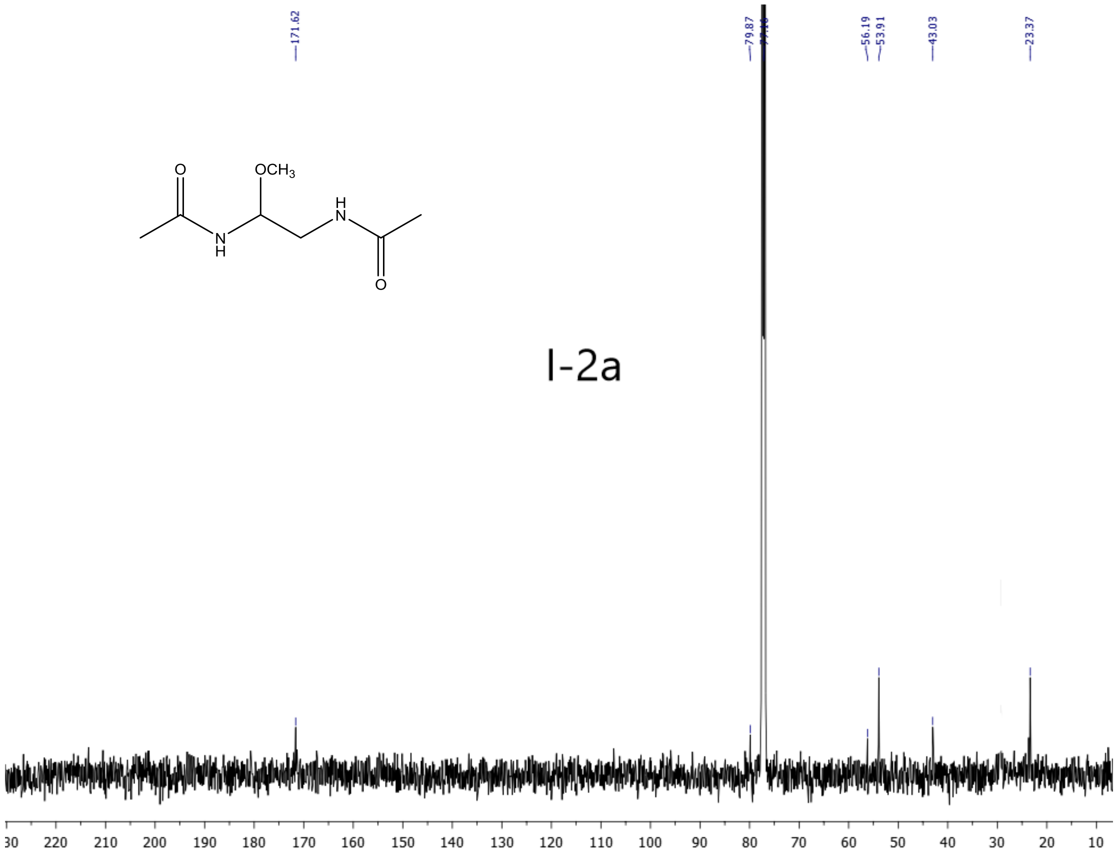

_N,N'_-(1-Methoxypropane-1,3-diyl)diacetamide (I-3a)

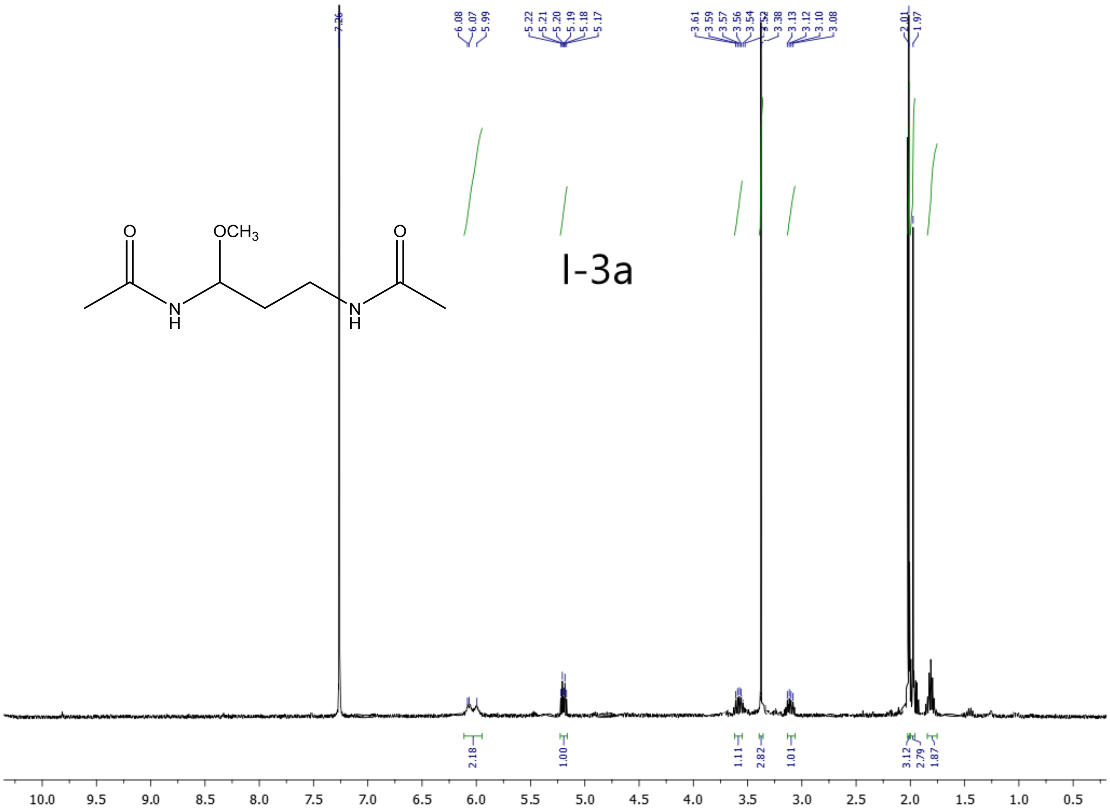

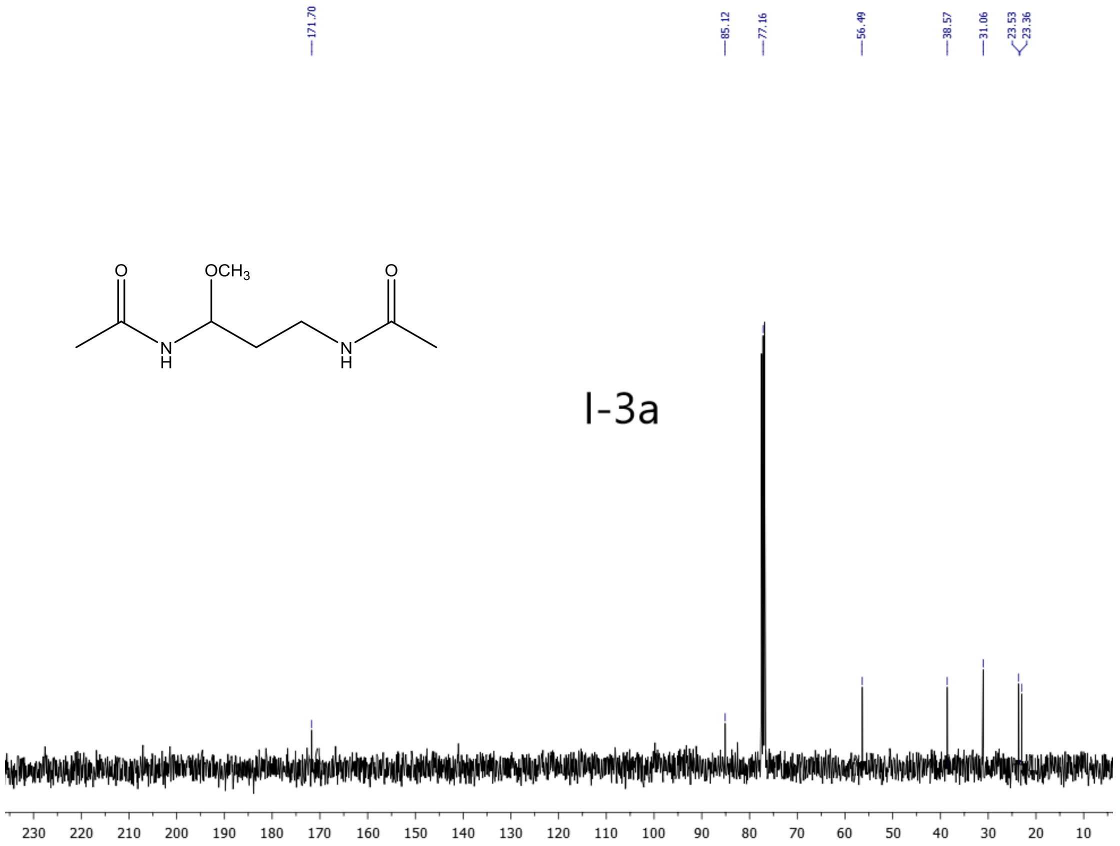

_N,N′_-(1,3-Dimethoxypropane-1,3-diyl)diacetamide (I-3b)

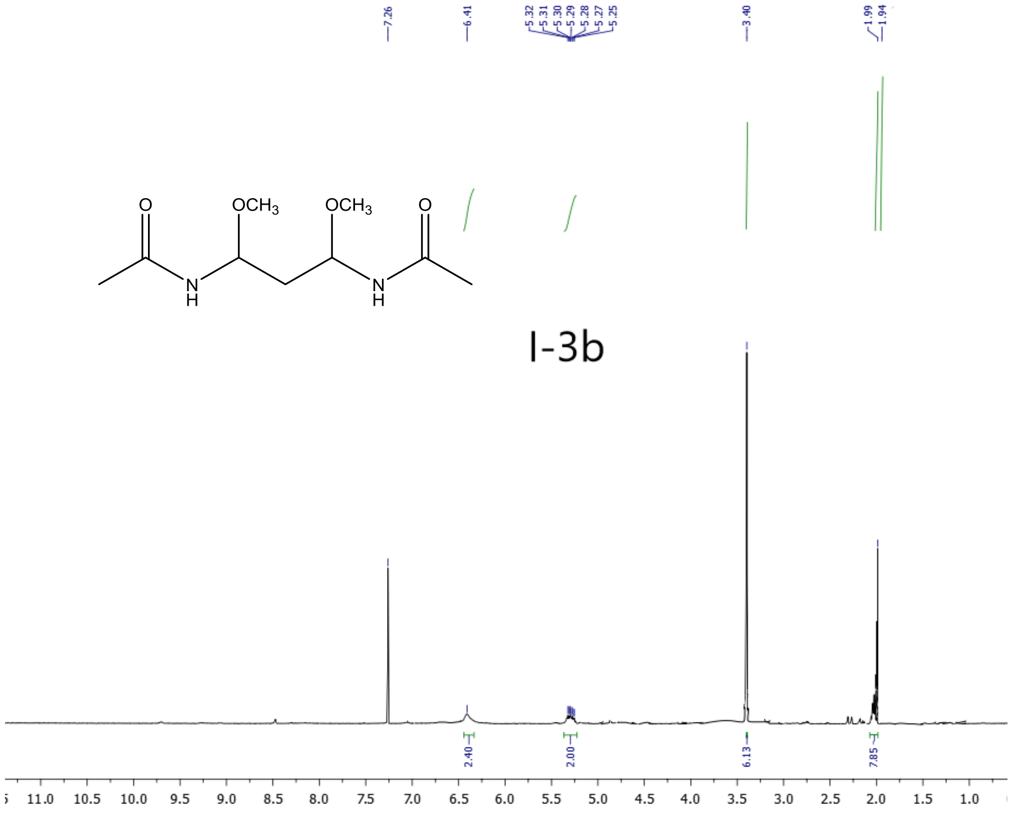

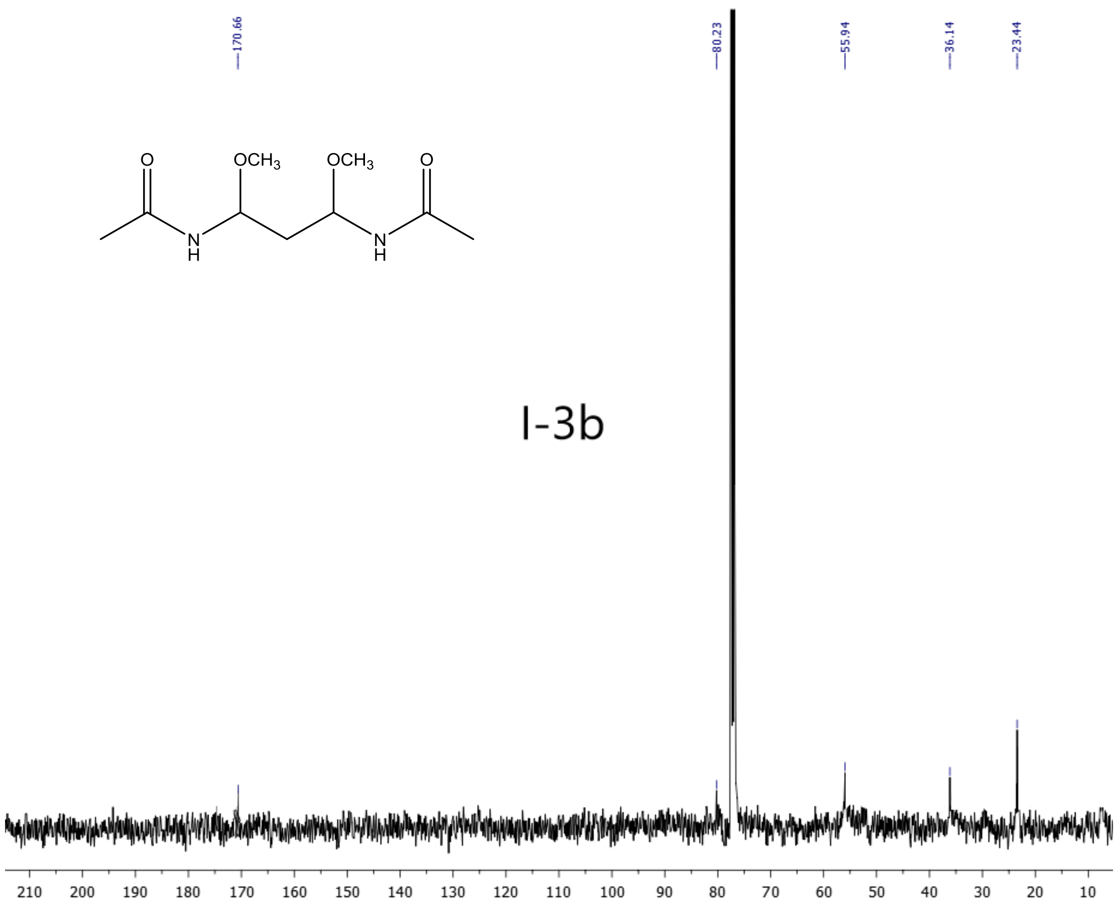

***N,N′*-(1-Methoxybutane-1,4-diyl)diacetamide (I-4a)**

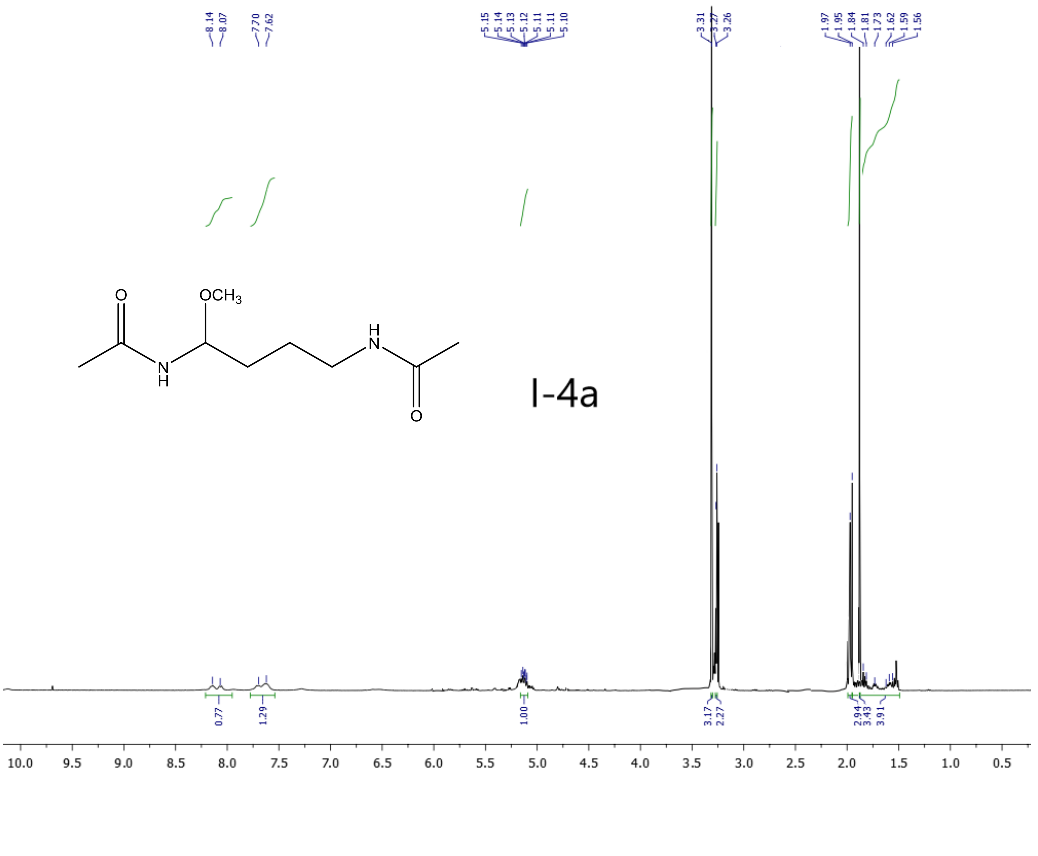

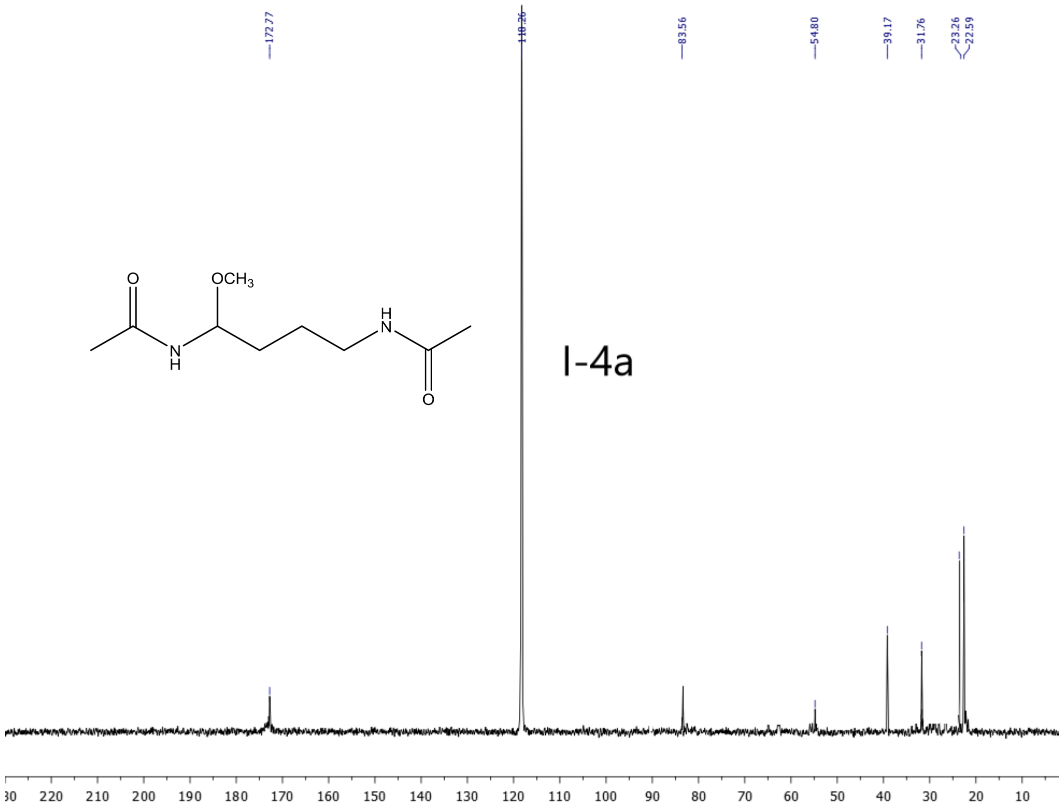

N,N'-(1,4-Dimethoxybutane-1,4-diyl)diacetamide (I-4b)

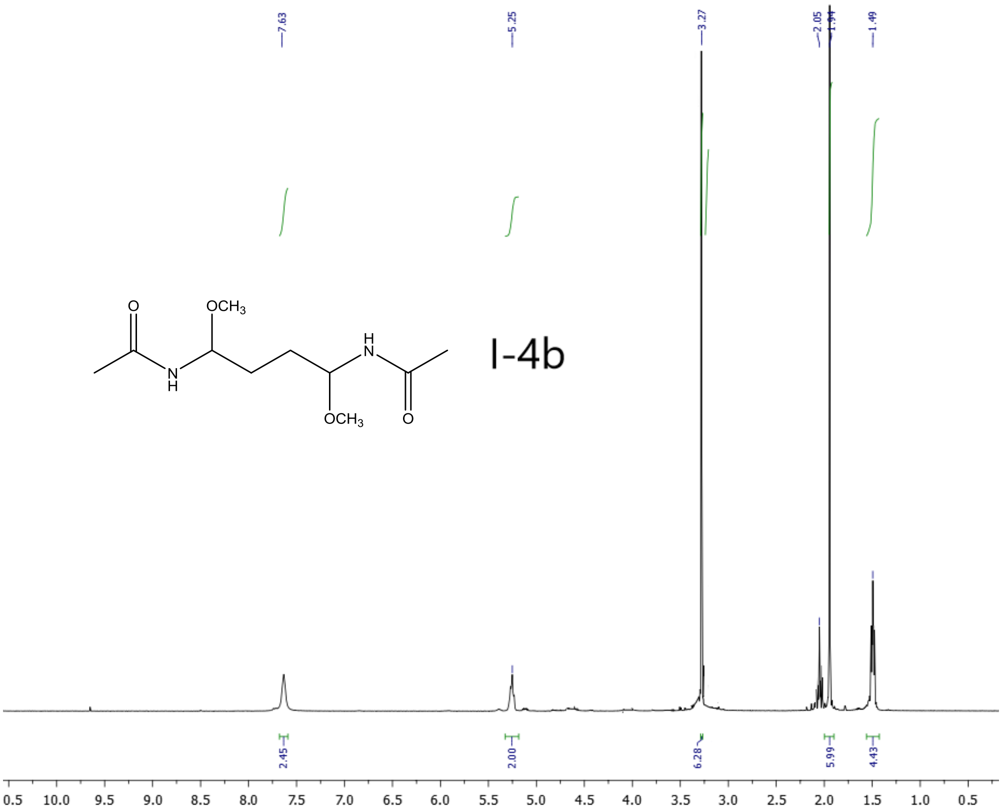

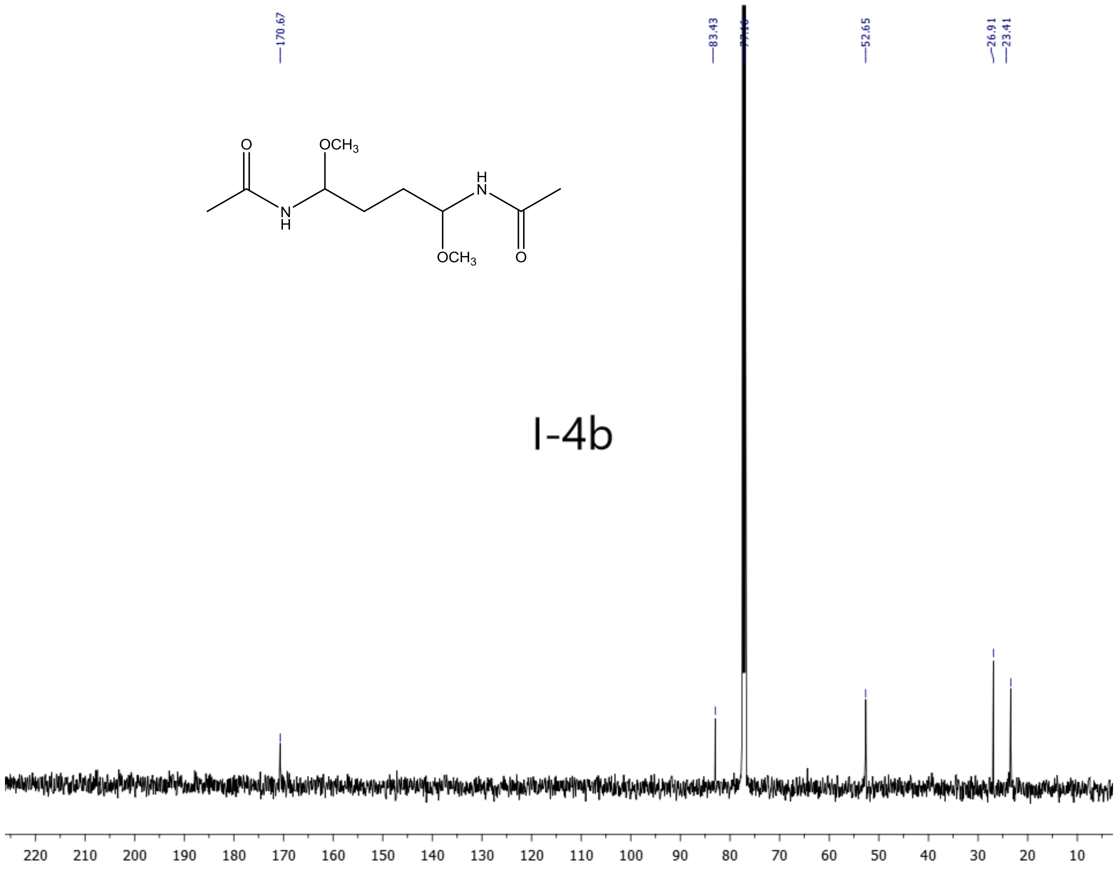

_N,N'_-(1-Methoxyethane-1,2-diyl)dibenzamide (II-2a)

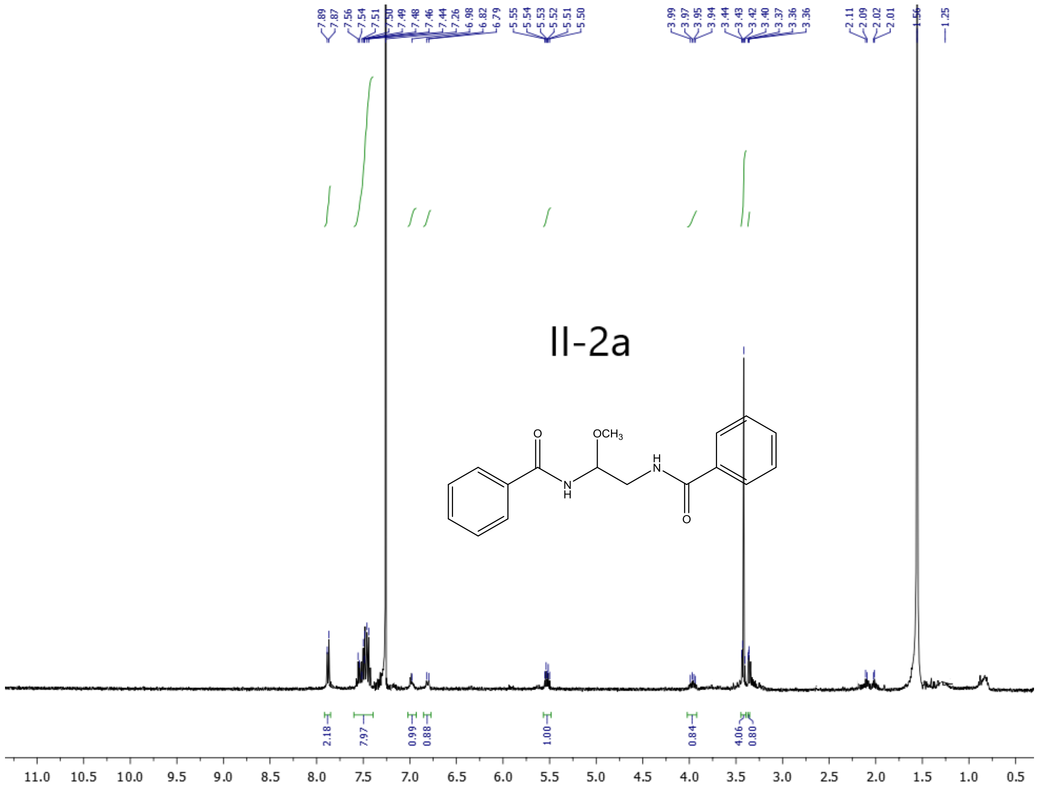

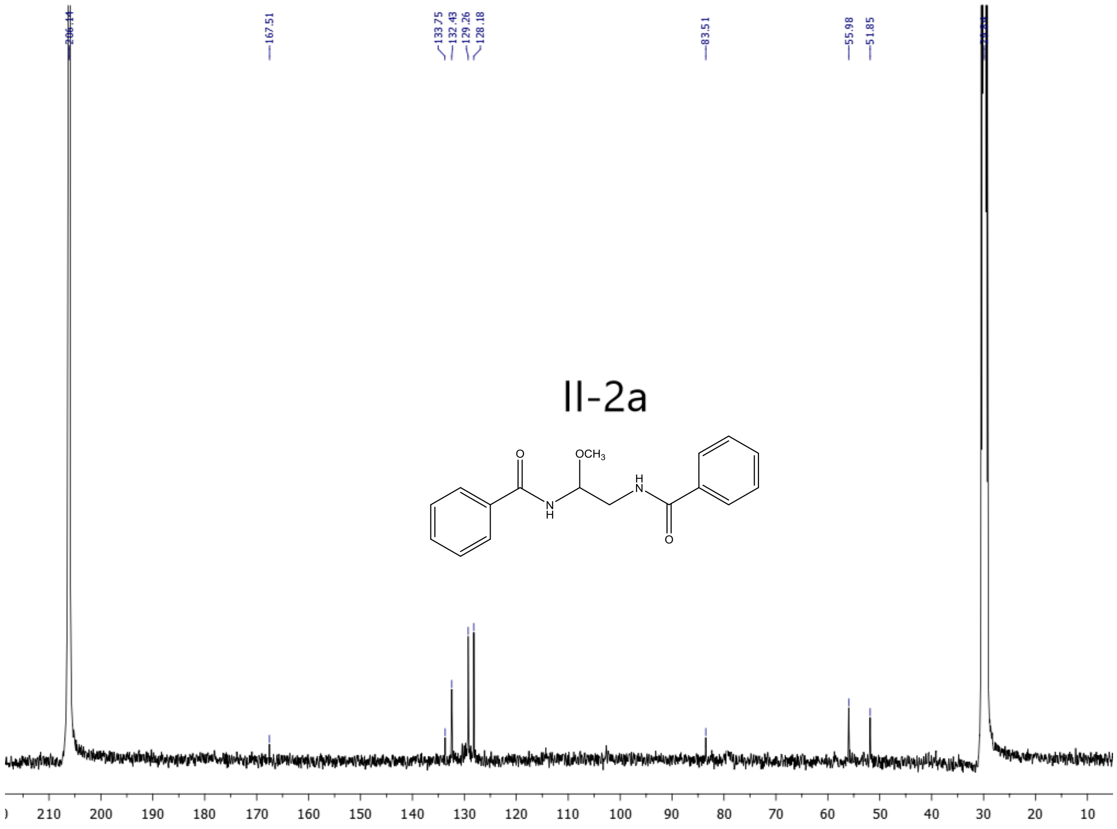

N,*N'*-(1-Methoxypropane-1,3-diyl)dibenzamide (II-3a)

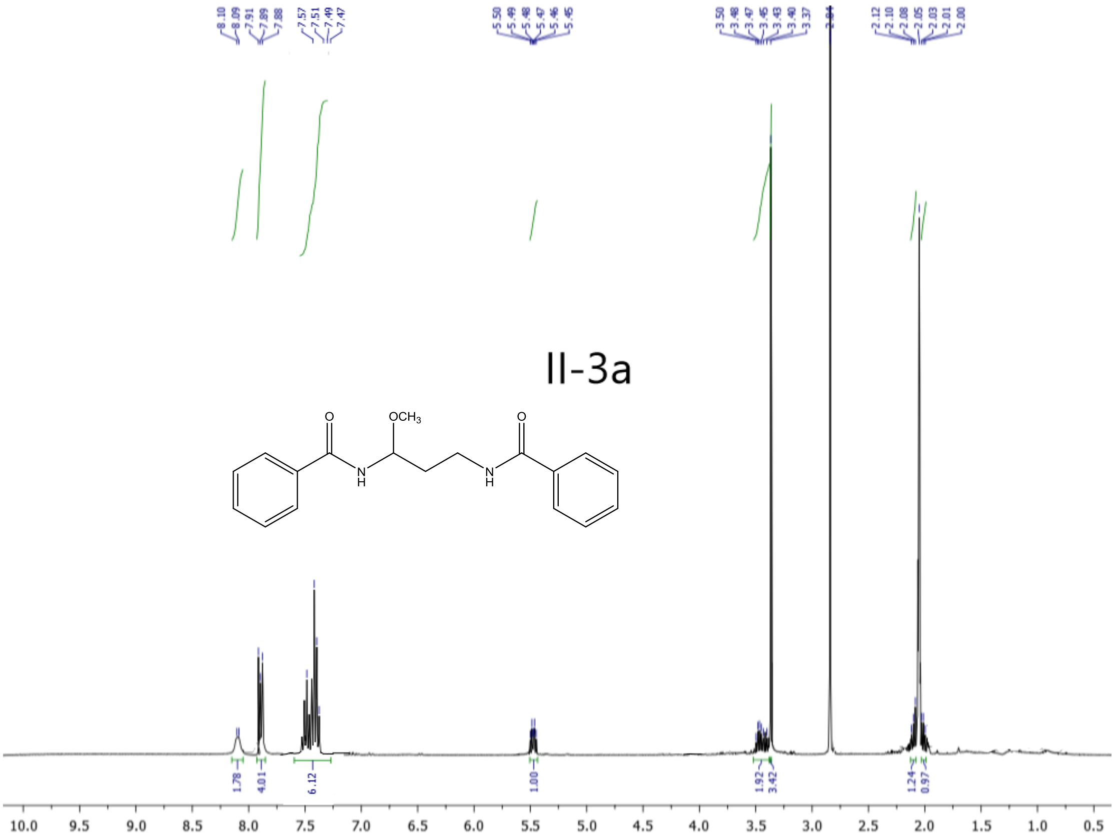

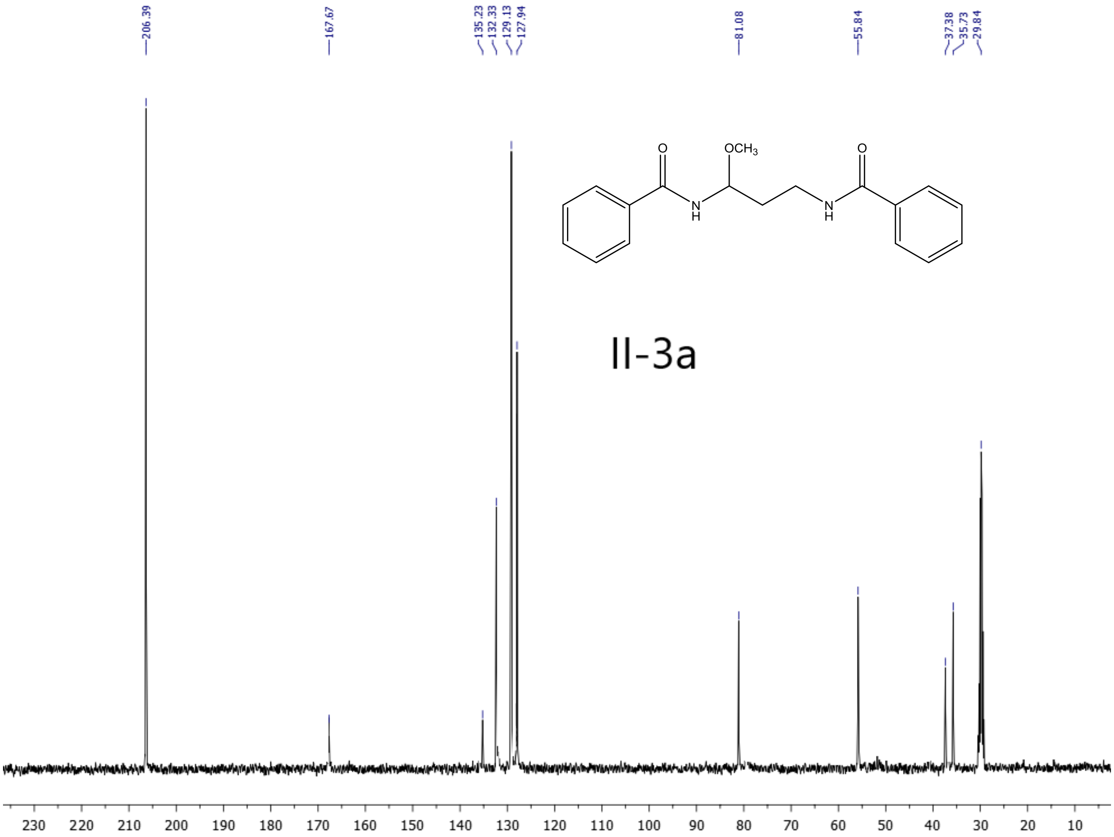

***N*,*N'*-(1,3-Dimethoxypropane-1,3-diyl)dibenzamide (II-3b)**

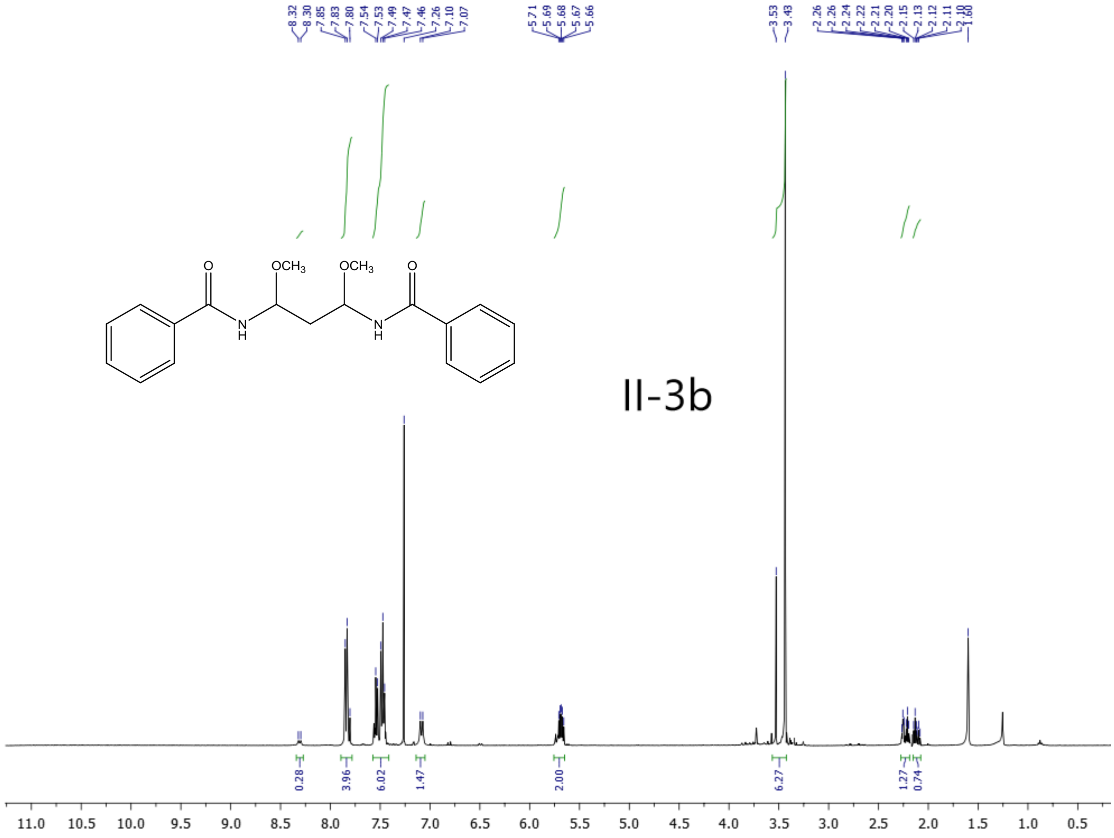

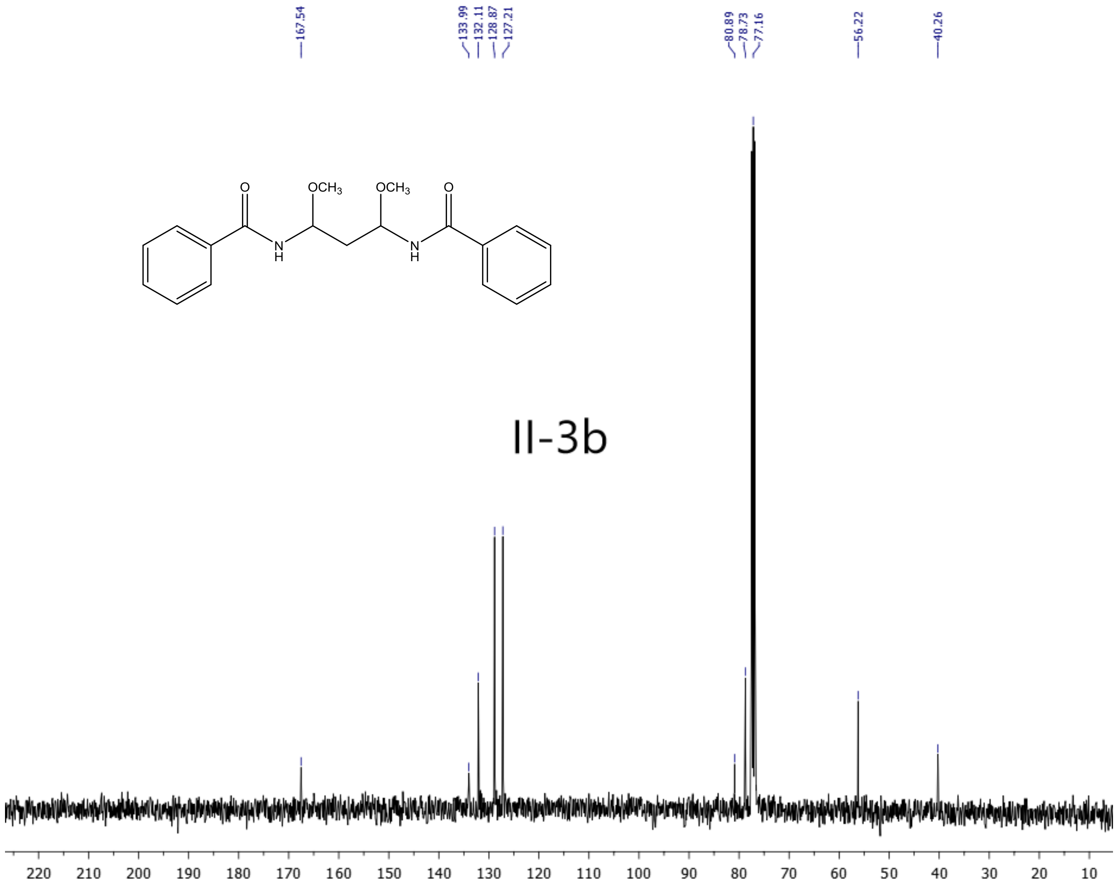

Methyl 3-benzamido-3-methoxy)propanoate (II-3c)

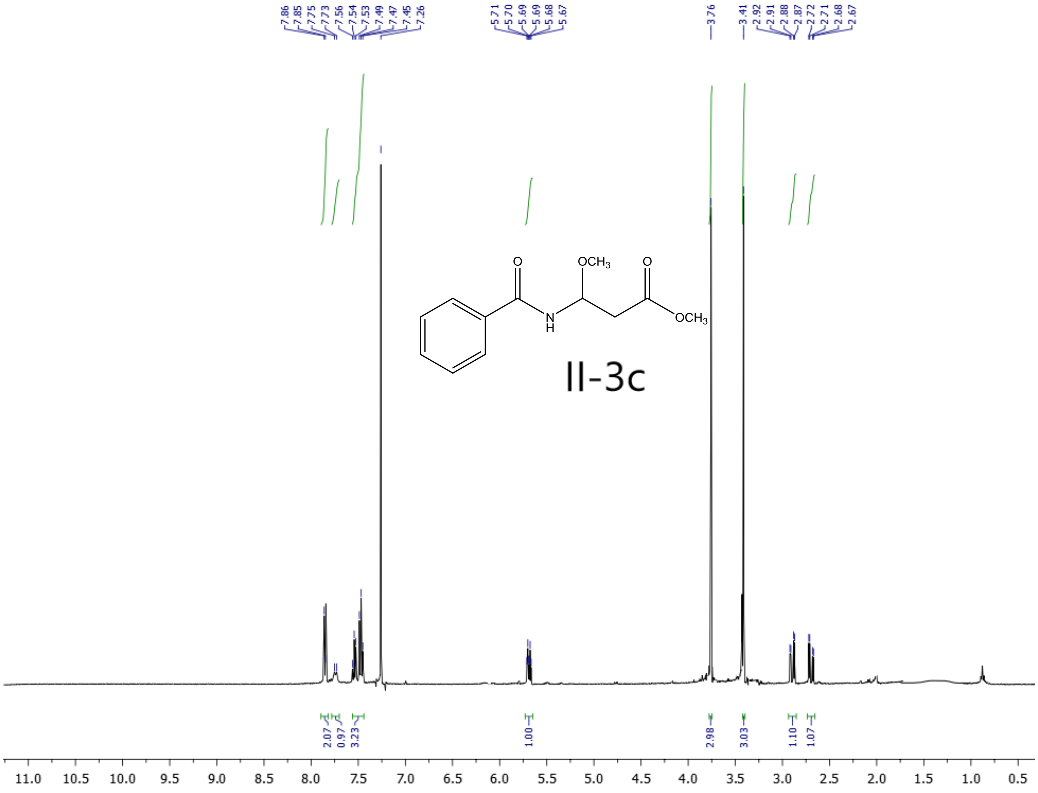

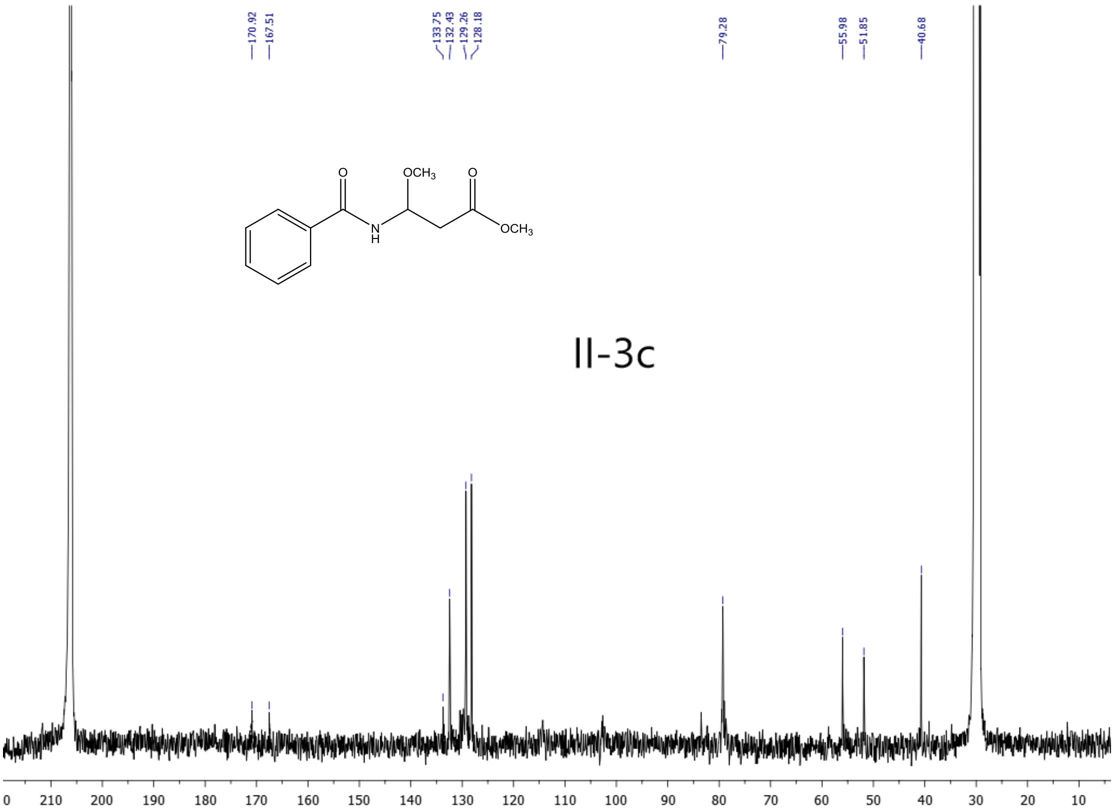

N,N'-(1-Methoxybutane-1,4-diyl)dibenzamide (II-4a)

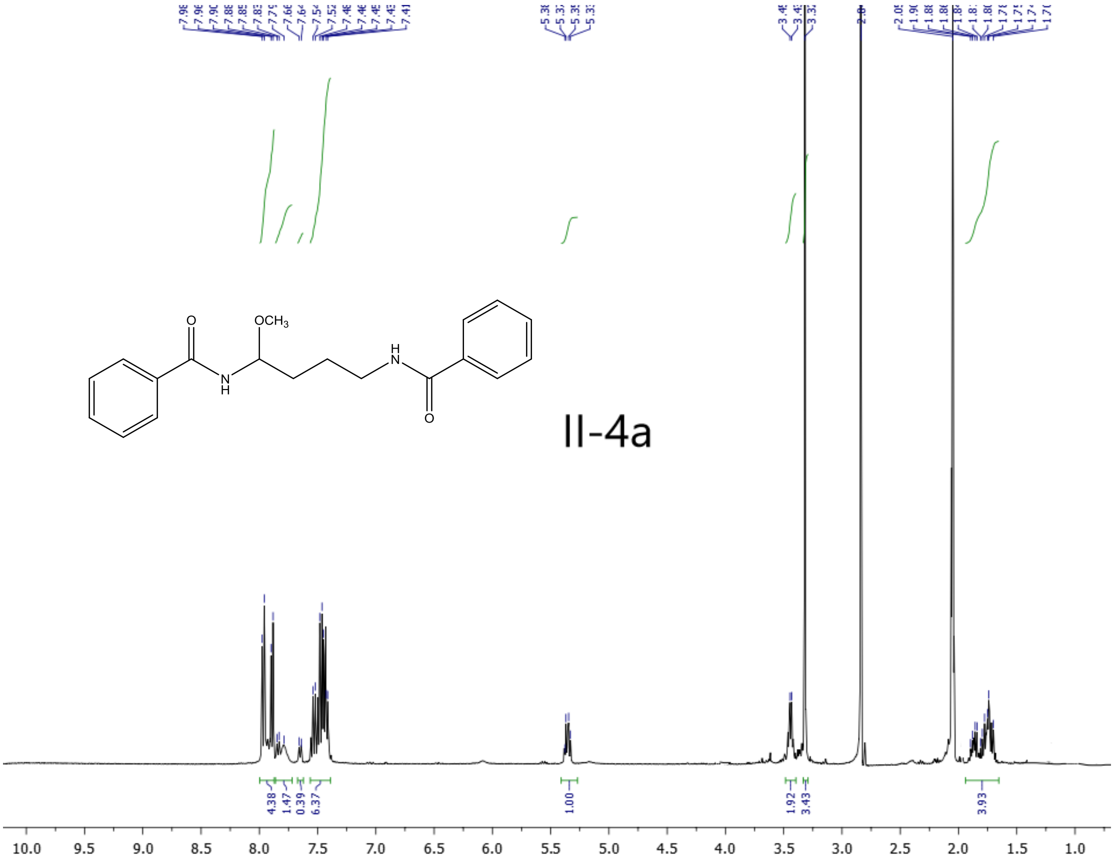

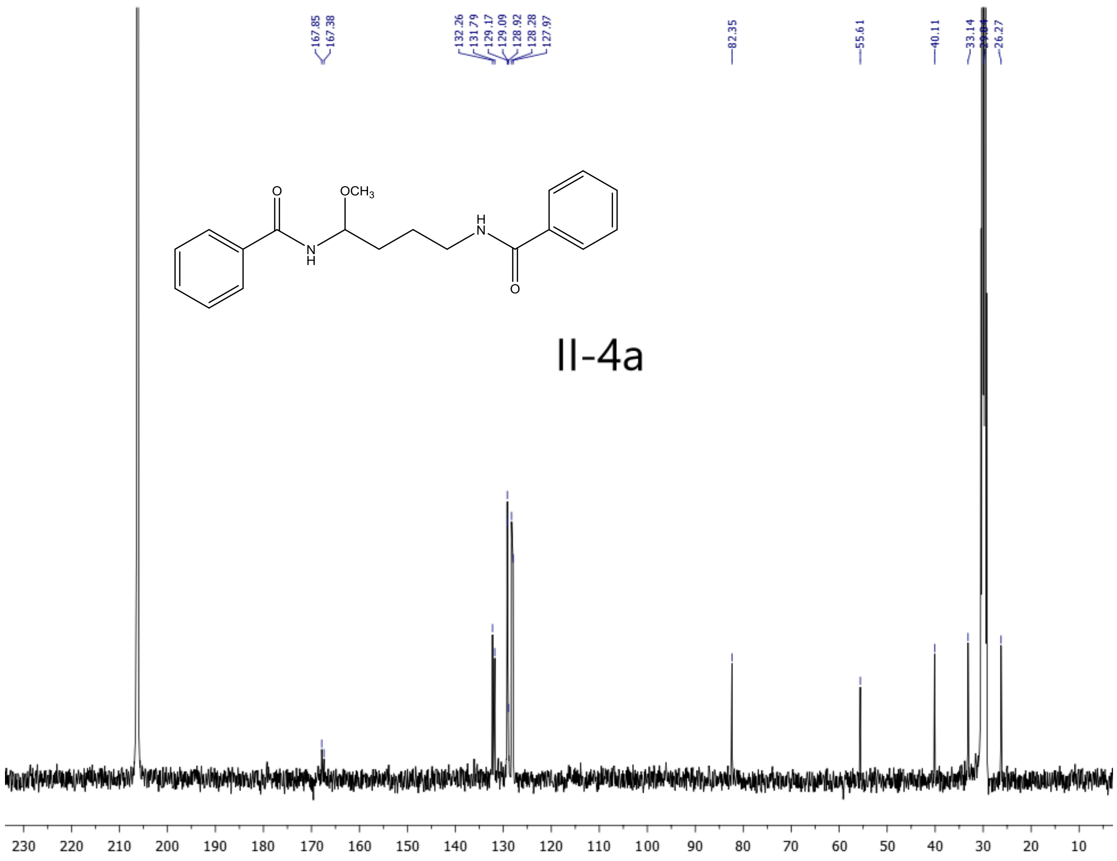

N,N'-(1,4-Dimethoxybutane-1,4-diyl)dibenzamide (II-4b)

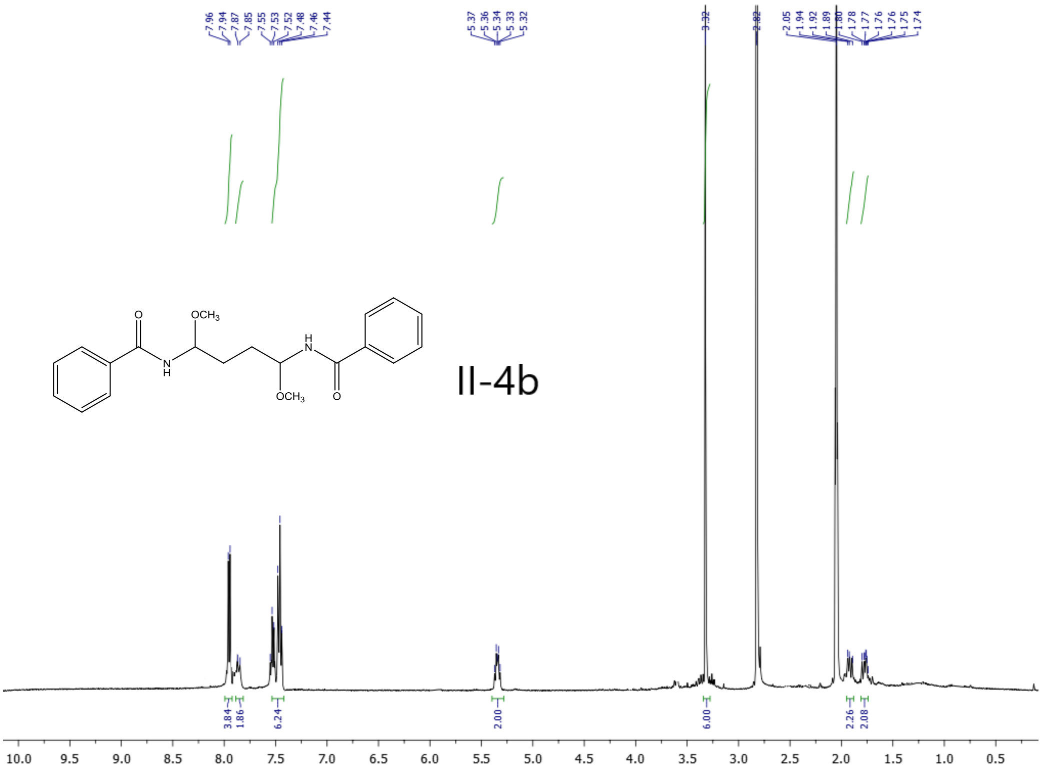

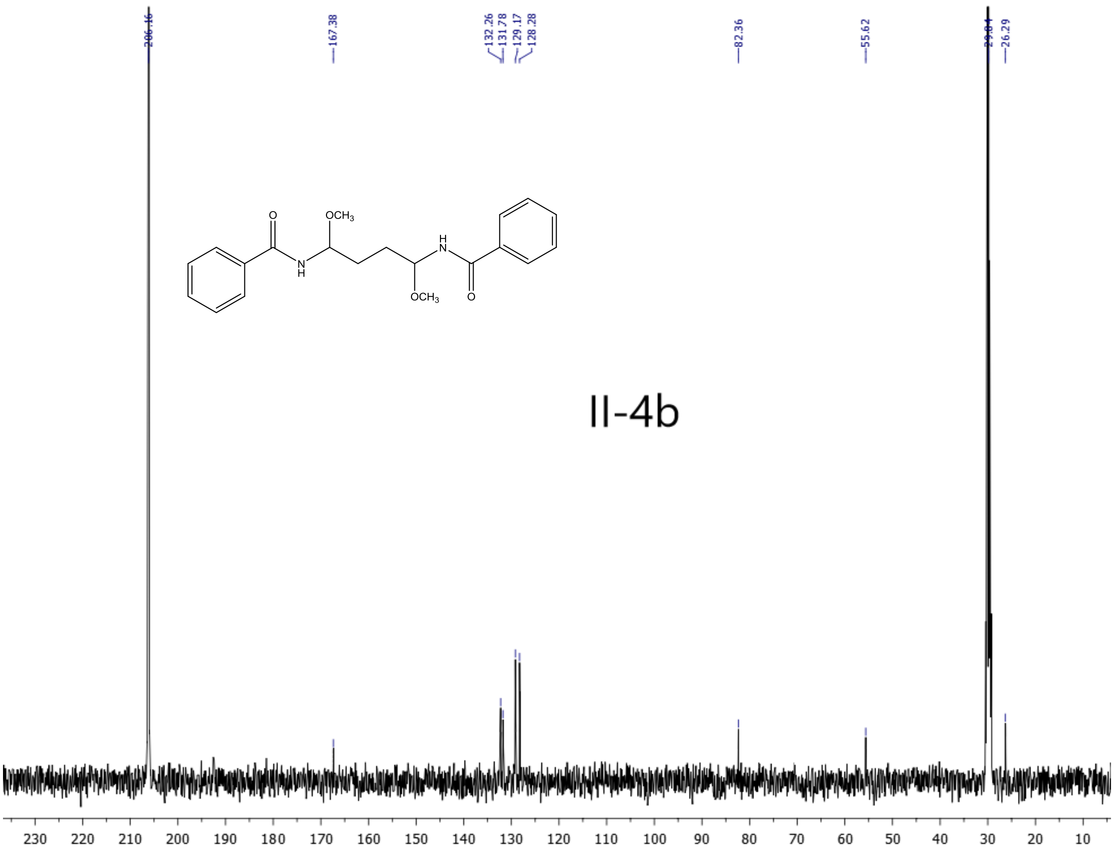

***N,N'*-(1-Methoxyethane-1,2-diyl)bis(4-methoxybenzamide) (III-2a)**

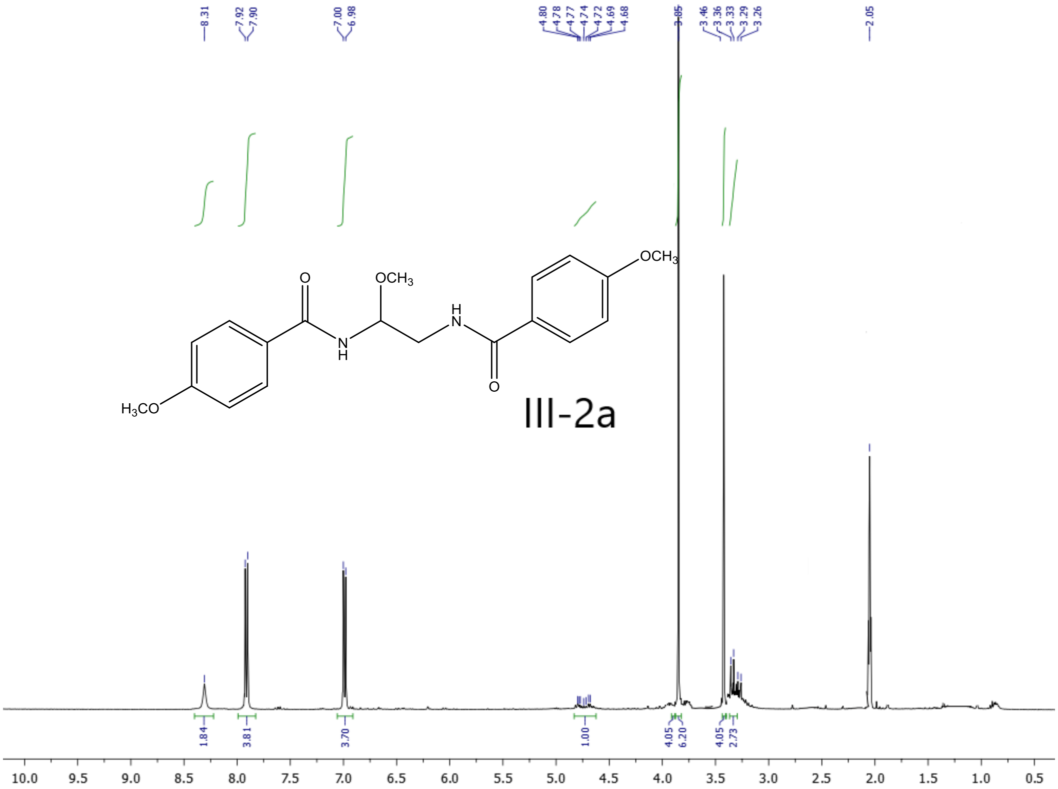

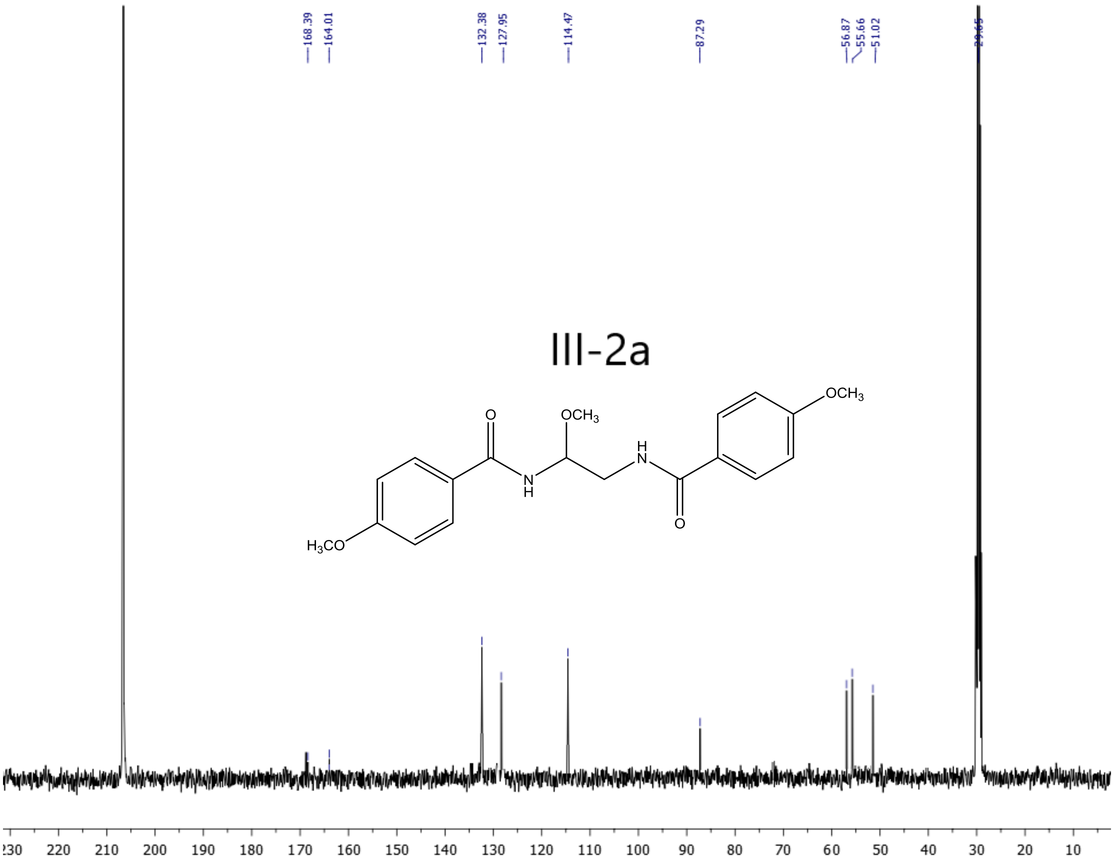

4-Methoxy-*N*-(methoxymethyl)benzamide (III-2f)

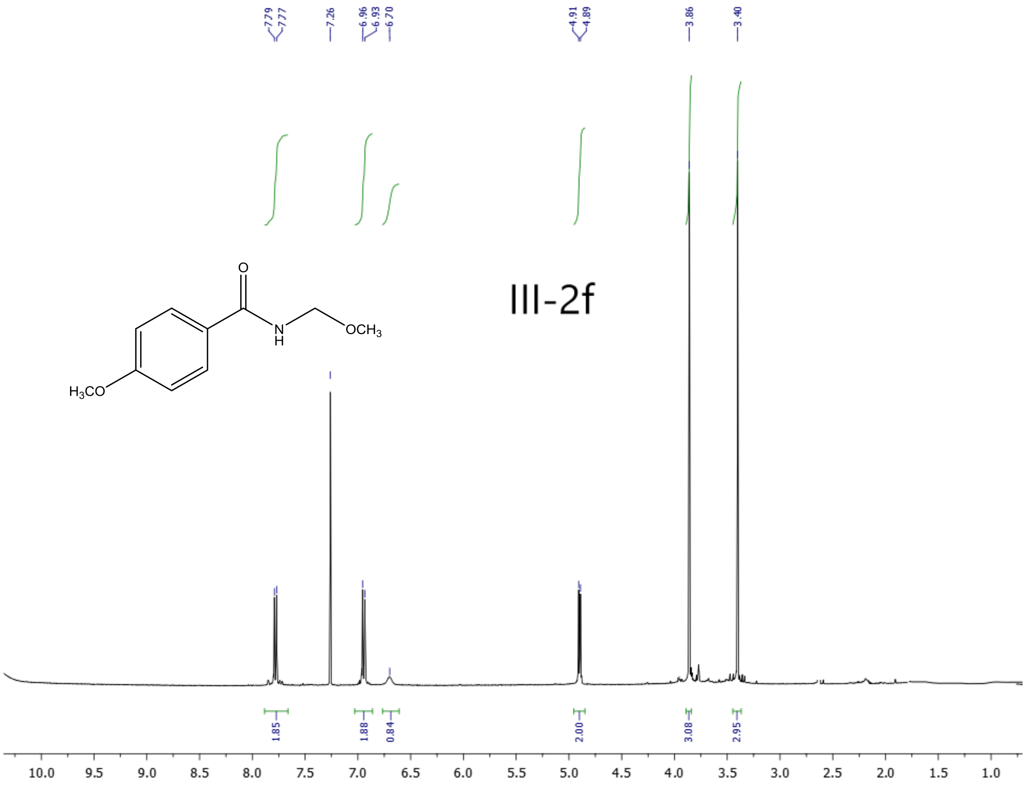

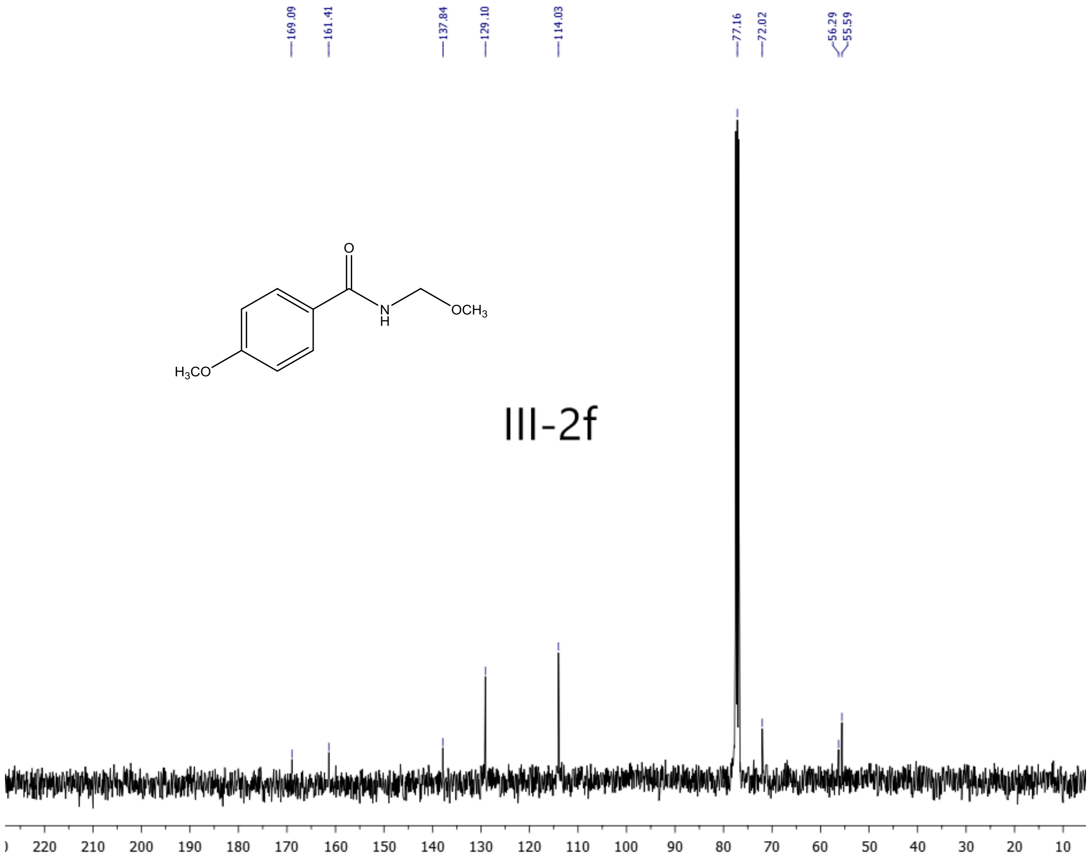

_N_-(Methoxymethyl)-4-nitrobenzamide (IV-2f)

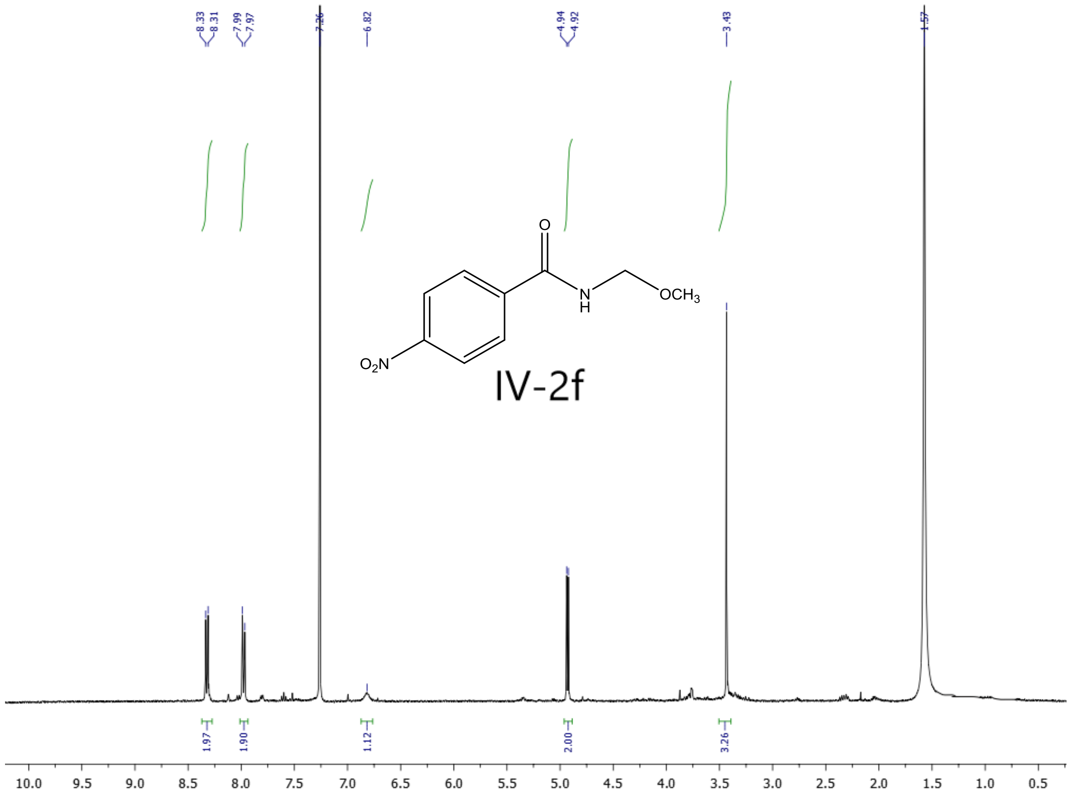

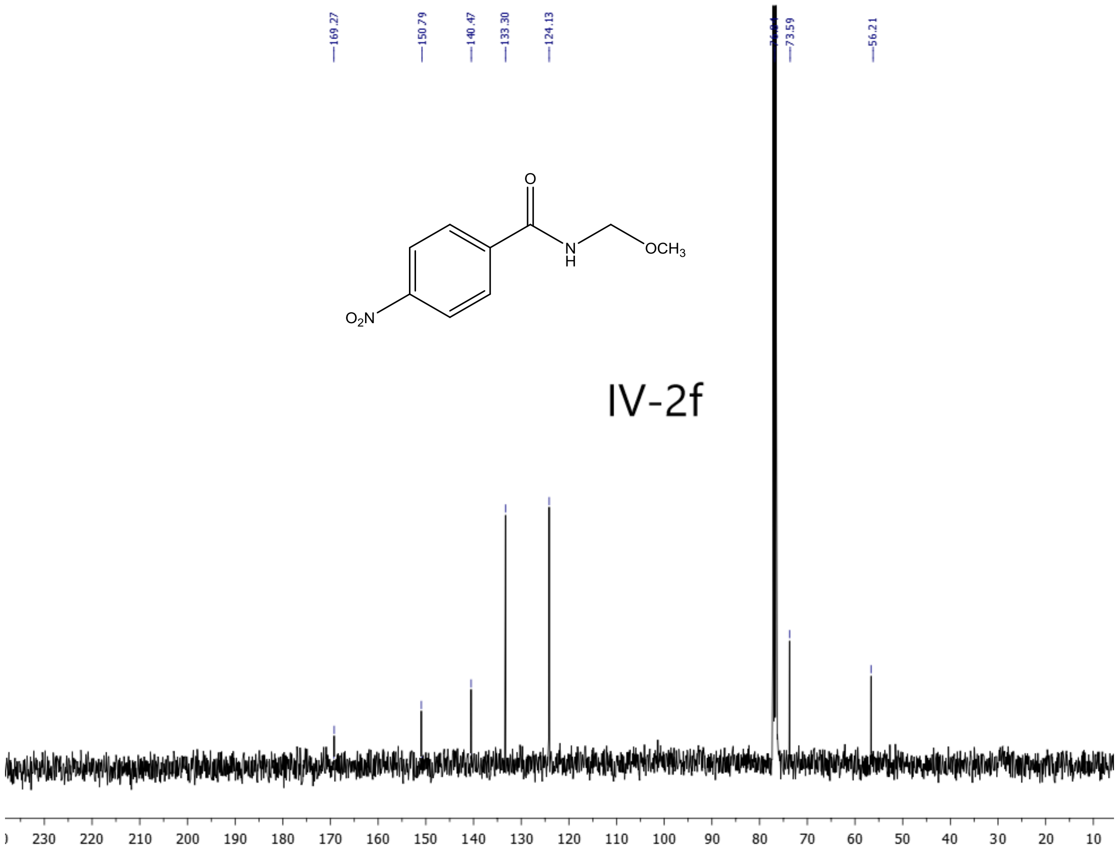

IV-2f

References:

[1] Lasne M.; Ripoll J.; Thuillier A. *Synopses*, 1982, 8, 214-215.

[2] Barnes I.; Solignas G. *Chem. Phys. Chem.*, 2010, 11, 3844-3857.

[3] Branchaud B.; Tsai P. *J. Org. Chem.*, 1987, 52, 5475-5478.

[4] Evans D.A.; Nagorny P.; R. Xu R. *Org. Lett.*, 2006, 24(8), 5669-5671.
